# The impact of anti-drug antibodies on drug concentrations and clinical outcomes in rheumatoid arthritis patients treated with adalimumab, etanercept, or infliximab: Results from a multinational, real-world clinical practice, non-interventional study

**DOI:** 10.1371/journal.pone.0175207

**Published:** 2017-04-27

**Authors:** Robert J. Moots, Ricardo M. Xavier, Chi Chiu Mok, Mahboob U. Rahman, Wen-Chan Tsai, Mustafa H. Al-Maini, Karel Pavelka, Ehab Mahgoub, Sameer Kotak, Joan Korth-Bradley, Ron Pedersen, Linda Mele, Qi Shen, Bonnie Vlahos

**Affiliations:** 1 Aintree University Hospital, University of Liverpool, Liverpool, United Kingdom; 2 Hospital de Clinicas, Porto Alegre, Brazil; 3 Tuen Mun Hospital, Hong Kong, People’s Republic of China; 4 Pfizer, Collegeville, Pennsylvania, United States of America; 5 Kaohsiung Medical University, Kaohsiung City, Taiwan; 6 Mafraq Hospital, Abu Dhabi, United Arab Emirates; 7 Institute of Rheumatology, Prague, Czech Republic; VU University Medical Center, NETHERLANDS

## Abstract

**Objective:**

To assess the incidence of anti-drug antibodies (ADA) in patients with rheumatoid arthritis (RA) treated with the TNF inhibitors etanercept (ETN), adalimumab (ADL), or infliximab (IFX), and determine the potential relationship with trough drug concentration, efficacy, and patient-reported outcomes.

**Methods:**

This multi-national, non-interventional, cross-sectional study (NCT01981473) enrolled adult patients with RA treated continuously for 6–24 months with ETN, ADL, or IFX. ADA and trough drug concentrations were measured by independent assays ≤2 days before the next scheduled dose. Efficacy measurements included Disease Activity Score 28-joint count (DAS28), low disease activity (LDA), remission, and erythrocyte sedimentation rate (ESR). Targeted medical histories of injection site/infusion reactions, serum sickness, and thromboembolic events were collected.

**Results:**

Baseline demographics of the 595 patients (ETN: n = 200; ADL: n = 199; IFX: n = 196) were similar across groups. The mean duration of treatment was 14.6, 13.5, and 13.1 months for ETN, ADL, and IFX, respectively. All ETN-treated patients tested negative for ADA, whereas 31.2% and 17.4% patients treated with ADL and IFX, respectively, tested positive. In ADL- or IFX-treated patients, those with ADA had significantly lower trough drug concentrations. There were negative correlations between trough drug levels and both CRP and ESR in ADL- and IFX-treated patients. DAS28-ESR LDA and remission rates were higher in patients without ADA. The rate of targeted medical events reported was low.

**Conclusion:**

ADA were detected in ADL- and IFX-treated but not ETN-treated patients. Patients without ADA generally showed numerically better clinical outcomes than those with ADA.

**Trial registration:**

This study was registered on www.ClinicalTrials.gov (NCT01981473).

## Introduction

Rheumatoid arthritis (RA) is a chronic, progressive, systemic inflammatory disease of unknown etiology characterized by chronic pain, joint destruction, and extra-articular co-morbidity [1]. The annual incidence of RA is estimated at 40/100,000 worldwide [1], and it is estimated to affect 1.3 million adults in the United States, corresponding to approximately 0.6% of the population [2].

Treatment with biologic tumor necrosis factor (TNF) inhibitors such as etanercept (ETN, a human soluble dimeric TNF receptor fusion protein), adalimumab (ADL, a fully human monoclonal antibody [mAb] against TNF), and infliximab (IFX, a mouse-human chimeric mAb against TNF) [3] has significantly reduced disease activity and improved quality of life in patients with RA who have not responded to conventional disease-modifying antirheumatic drug (DMARD) therapy [4]. All three TNF inhibitors are proteins and, therefore, are inherently immunogenic. Since treatment requires continued dosing for efficacy [5,6], there is a potential for patients to develop anti-drug antibodies (ADA) over time [7–9]. The presence of ADA can cause serum drug levels to drop to sub-therapeutic levels [10–12], or neutralize the drug [12–14], resulting in loss of clinical response [10–14]. ADA can also contribute to injection site and infusion reactions, thromboembolic events, and serum sickness, thereby raising safety concerns [14–16].

Previous studies have shown that up to 44% of patients treated with IFX [11,15,17] reported having ADA within the first 6 months of treatment [15]. ADA have also been reported for patients treated with ADL [11,12], with 19% of patients exhibiting ADA within the first 6 months of treatment and increasing to 28% within 3 years [12]. By contrast, studies of patients treated with ETN have reported an incidence of ADA in 0–7% of patients; when present, they have been generally transient and non-neutralizing [9,13,18,19]. A meta-analysis of 17 studies evaluating the immunogenicity of TNF inhibitors reported that no ADA were detected in response to ETN treatment [13].

The existing reports on the incidence of ADA in response to treatment with TNF inhibitors are primarily based on data from clinical trials of drug treatment with a single therapeutic agent. There is no study that measured ADA concomitantly for multiple TNF inhibitors. The data on ADA and their impact on serum drug concentrations and clinical outcomes were generated by different investigators using different laboratories and assay methods, which makes it harder to compare the results between various studies.

The aim of this single, non-interventional, cross-sectional study was to assess the immunogenicity of ETN, ADL, and IFX and its impact on serum trough drug concentration and efficacy in patients with RA when used in a routine real-world, clinical practice setting. All samples were handled the same way and analyzed using the same validated commercially available assays in a single independent laboratory.

## Patients and methods

### Patients inclusion and exclusion criteria

**Fig 1:**
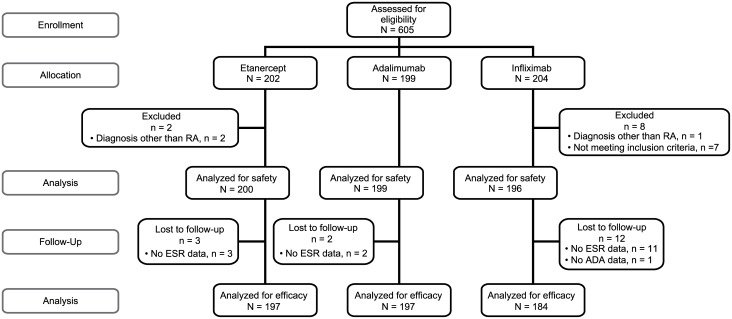
Patient disposition.

All adult (≥18 years of age) patients with RA (American College of Rheumatology 1987 criteria) undergoing continuous treatment with ETN, ADL, or IFX for a minimum of 6 months and a maximum of 24 months prior to the study assessment visit were eligible for this study. Exclusion criteria included the following: treatment with a biosimilar or investigational ETN, ADL, or IFX within 6 months of the study assessment visit; treatment with any other investigational drug within 3 months or five half-lives of the drug, whichever was longer, of the study assessment visit; having a history of any medical condition that would interfere with efficacy or other assessments (e.g., fibromyalgia, lupus); or inability to provide informed consent (S1 File). Patient disposition is given in Fig 1. The study was approved by local ethics committees (Comité Independiente de Ética Para Ensayos en Farmacología Clínica Fundación de Estudios Farmacológicos y de Medicamentos, J.E. Uriburu 774, Piso 1° (C1027AAP), Buenos Aires, Argentina; Comité de Revisión Institucional del Hospital Británico, Perdriel 74 (C1280AEB), Buenos Aires, Argentina; Comité de Ética CAICI–CIAP, Rodriguez 1198 (2000), Rosario, Santa Fe, Buenos Aires, Argentina; Comité de Ética de Protocolos de Investigación del Hospital Italiano de Buenos Aires Juan D. Perón 4190 (1181), C.A.B.A., Buenos Aires, Argentina; Comité de Ética San Isidro Av. del Libertador 16958, San Isidro, Buenos Aires, Argentina; Comité de Ética en Investigación DIM Clínica Privada, Espora 18, (B1704FAB), Ramos Mejía, Buenos Aires, Argentina; Bellberry Human Research Ethics Committee, 129 Glen Osmond Road, Eastwood SA 5063, Australia; Ethics Committee for Multicenter Trials, 5 St. Nedelya Square, 1000 Sofia, Bulgaria; Hacettepe Üniversitesi (Hacettepe University), Klînîk Araştirmalar Etik Kurulu (Clinical Trials Ethics Committee), 06100 Altindağ / Ankara, Turkey; Ministry of Health, Republic of Turkey T.C. Sağlik Bakanliği (Ministry Of Health) Türkiye İlaç ve Tıbbi Cihaz Kurumu (Turkish Drug and Medical Device Institution) Söğütözü Mahallesi, 2176. Sokak No:5 06520 Çankaya/Ankara, Turkey; Schulman Associates IRB, 4445 Lake Forest Drive Suite 300, Cincinnati, OH 45242, United States; and Western Institutional Review Board, 1019 39th Avenue SE Suite 120, Puyallup, WA 98374–2115, United States) and all patients provided written consent according to the Declaration of Helsinki.

### Study design

**Fig 2:**
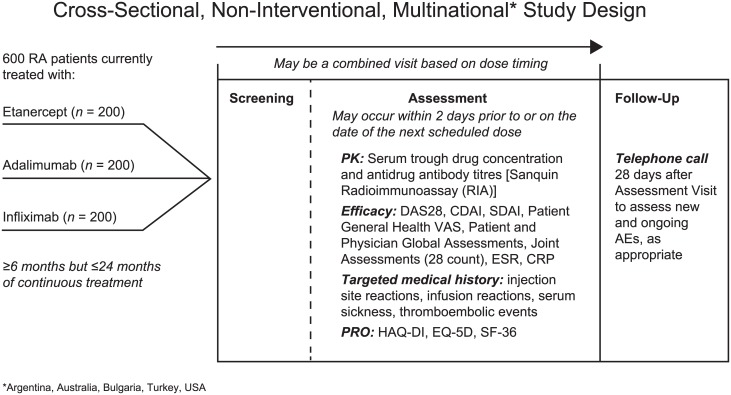
Study design. RA = rheumatoid arthritis; PK = pharmacokinetics; DAS28 = Disease Activity Score based on a 28-joint count; CDAI = Clinical Disease Activity Index; SDAI = Simplified Disease Activity Index; VAS = visual analog scale; ESR = erythrocyte sedimentation rate; CRP = C-reactive protein; PRO = patient- reported outcome; HAQ-DI = health assessment questionnaire-disability index; EQ-5D = EuroQol-5 Dimensions; SF-36 = Short-Form Health Survey; AE = adverse event. * Argentina, Australia, Bulgaria, Turkey, USA.

In this multicenter, non-interventional, cross-sectional study (Fig 2), approximately 600 patients from Argentina, Australia, Bulgaria, Turkey, and the United States treated with ETN, ADL, and IFX continuously for 6 to 12 months in a real-world clinical setting were to be enrolled, with approximately 200 patients associated with each RA treatment. This sample was sufficient to provide >95% power to detect a difference of 12% in the proportion of patients positive for ADA between the group of patients treated with a soluble receptor TNF inhibitor (ETN) and the group of patients treated with anti-TNF monoclonal antibodies (ADL and IFX) using a χ^2^ test with continuity correction, an alpha of 0.05, and an attrition rate of 15%.

Data and laboratory samples were collected in a single visit for the assessment of treatment history and dosing, disease status, patient-reported outcomes, serum trough drug concentration, ADA concentration, and targeted medical history, which included injection site reactions, infusion reactions, serum sickness, and thromboembolic events.

All assays were conducted at the same central laboratory (Sanquin, Amsterdam, The Netherlands). Drug concentrations were analyzed using validated enzyme-linked immunosorbent assay methods with lower limits of quantitation of 0.10 μg/mL for ETN and IFX and 0.03 μg/mL for ADL. ADA titers were analyzed using validated radioimmunoassay methods. Patients having ADA concentrations >12 antibody units/mL were classified as positive.

### Endpoints

The primary pre-specified endpoint of this study was the proportion of patients testing positive for ADA among those treated with ETN versus the pooled data for patients treated with ADL or IFX. Secondary endpoints included efficacy measures such as Clinical Disease Activity Index (CDAI), Simplified Disease Activity Index (SDAI), Disease Activity Score based on 28-joint count (DAS28), erythrocyte sedimentation rate (ESR), and DAS28-C-reactive protein (CRP) scores, and health outcomes measures including the Health Assessment Questionnaire–Disability Index (HAQ-DI), 36-Item Short-Form Health Survey (SF-36), and EuroQol-5 Dimensions (EQ-5D) questionnaires. Additional endpoints comparing patients who were ADA-positive with those who were ADA-negative included the proportion of patients with low disease activity (LDA), defined as a DAS28-ESR score ≤3.2; serum trough drug concentrations for ETN, ADL, and IFX; HAQ-DI scores; and correlations of ADA titers with efficacy measures and serum trough drug concentrations.

### Safety

Safety was evaluated at screening, at the assessment visit, and during the follow-up telephone call by recording adverse events (AEs), serious AEs (SAEs), and targeted medical events (injection site reactions, infusion reactions, serum sickness, and thromboembolic events).

### Statistics

Continuous data were characterized by arithmetic mean, standard deviation (SD), median, minimum, maximum, and number of observations. Categorical data were summarized using counts and percentages. Comparisons between the proportions of patients with certain characteristics in the subset of patients with detectable ADA versus those without ADA were performed using Fisher’s exact tests. The comparison between patients with detectable ADA versus those without ADA with regard to continuous variables (such as serum trough drug concentration, efficacy scores, and health outcomes measures) was performed using t-tests or analysis of variance (ANOVA). Correlations between continuous variables were evaluated with Spearman’s correlation coefficients.

## Results

### Patients

A total of 605 patients were enrolled in the study, of which 595 were eligible for analysis: ETN, n = 200; ADL, n = 199; and IFX, n = 196. Baseline characteristics were similar across the three treatment groups, including age, proportion of females, ethnicity, and symptom duration (Table 1). The mean duration of treatment was similar for the three drugs. The proportions of patients treated for 6–12 months were 34.0%, 49.2%, and 49.5% in the ETN, ADL, and IFX groups, respectively. The corresponding proportions were 37.5%, 25.1%, and 28.6% for patients treated for 12–18 months, and 28.5%, 25.6%, and 21.9% for patients treated for 18–24 months. The treatment doses of all three drugs were within the approved prescribing range for each drug (Table 1).

**Table 1:** Patient characteristics.

Parameter	ETN (n = 200)	ADL (n = 199)	IFX (n = 196)	Total (N = 595)
Age, mean (SD), years	56.5 (13.37)	54.3 (12.95)	60.7 (13.01	57.1 (13.36)
Female, n (%)	155 (77.5)	162 (81.4)	157 (80.1)	474 (79.7)
Race, n (%)				
White	184 (92.0)	179 (89.9)	176 (89.8)	539 (90.6)
Other	8 (4.0)	12 (6.0)	7 (3.6)	27 (4.5)
Black	8 (4.0)	7 (3.5)	10 (5.1)	25 (4.2)
Asian	0	1 (0.5)	3 (1.5)	4 (0.7)
Symptom duration, mean (SD), years	10.8 (10.67)	9.3 (8.43)	10.0 (10.11)	10.0 (9.78)
Treatment interval dose, mean (SD)	49.9 (1.77) mg	42.1 (8.82) mg	6.4 (3.71) mg/kg	
Drug dose, min–max	25.0–50.0 mg	37.3–80.0 mg	2.0–20.0 mg/kg	
Median drug dose	50.0 mg	40.0 mg	5.0 mg/kg	
Duration of treatment, mean (SD), months	14.6 (5.35)	13.5 (5.52)	13.1 (5.36)	13.7 (5.44)
6 to <12 months, n (%)	68 (34.0)	98 (49.2)	97 (49.5)	263 (44.2)
12 to <18 months, n (%)	75 (37.5)	50 (25.1)	56 (28.6)	181 (30.4)
18 to ≤24 months, n (%)	57 (28.5)	51 (25.6)	43 (21.9)	151 (25.4)
Prior biologic treatments, n (%)	38 (19.0)	40 (20.1)	82 (41/8)	
Current MTX, n (%)	123 (61.5)	140 (70.4)	125 (63.8)	
Current other DMARDs, n (%)	41 (20.5)	37 (18.6)	59 (30.1)	
Current corticosteroids, n (%)	65 (32.5)	64 (32.2)	78 (39.8)	
Current NSAIDs, n (%)	66 (33.0)	58 (29.1)	59 (30.1)	

ETN = etanercept; ADL = adalimumab; IFX = infliximab; NSAID = nonsteroidal anti-inflammatory drug; SD = standard deviation; MTX = methotrexate.

### Immunogenicity

**Fig 3:**
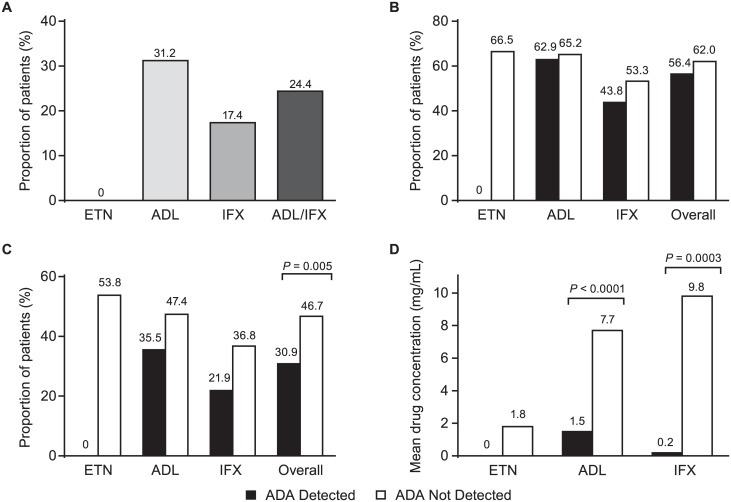
Proportion of patients (A) with ADA by treatment, (B) achieving LDA by treatment and ADA status, (C) achieving remission by treatment and ADA status, and (D) mean drug concentration by treatment. ADA = antidrug antibodies; LDA = low disease activity; ETN = etanercept; ADL = adalimumab; IFX = infliximab.

ADA were not detected in any patient treated with ETN compared with 96/394 (24.4%) patients treated with monoclonal anti-TNF antibodies (ADL and IFX combined; *P* < 0.0001; Fig 3A). There were 62/199 (31.2%) patients treated with ADL and 34/195 (17.4%) patients treated with IFX testing positive for ADA. The incidence of ADA in ADL- and IFX-treated groups was 34.7% and 26.8%, respectively, in patients treated for 6–12 months; 32.0% and 9.1%, respectively, in patients treated for 12–18 months; and 23.5% and 7.0%, respectively, in patients treated for 18–24 months. The incidence of ADA with respect to duration of treatment was statistically significant for only the IFX-treated group (*P* = 0.0014). The incidence of ADA in the ADL- and IFX-treated groups was 29.3% and 14.5%, respectively, for patients currently on methotrexate (MTX), and 35.6% and 22.5%, respectively, for patients currently not on MTX. The differences in proportion of patients with detectable ADA between those who were and who were not receiving MTX were not statistically significant for either the ADL- or IFX-treatment groups.

### Efficacy

Efficacy data were available for 578 of the 595 patients eligible for analysis; ESR data, necessary for determining the DAS28-ESR score, were not available for 16 patients (ETN, n = 3; ADL, N = 2; IFX, n = 11); ADA data, necessary for analysis of efficacy by ADA, were not available for one patient (IFX). Pooled data from all three TNF inhibitors showed that of a total of 353/578 (61.1%) patients who were in LDA, a numerically greater proportion (*P* = 0.3552) of patients had no detectable ADA (300/484; 62.0%) versus those with detectable ADA (53/94; 56.4%). When only patients treated with ADL and IFX were pooled (excluding patients treated with ETN as none of them had detectable ADA), the proportion of patients without detectable ADA who were in LDA was 169/287 (58.9%) and similar to that among patients with detectable ADA. Of the 200 patients treated with ETN, 131 (66.5%) were in LDA (Fig 3B). Among patients treated with ADL, overall 127/197 (64.5%) were in LDA, of which 39/62 (62.9%) had detectable ADA and 88/135 (65.2%) had no detectable ADA(*P* = 0.7516). Of the patients treated with IFX, overall 95/184 (51.6%) were in LDA, of which 14/32 (43.8%) had detectable ADA and 81/152 (53.3%) had no detectable ADA (*P* = 0.3387).

**Fig 4:**
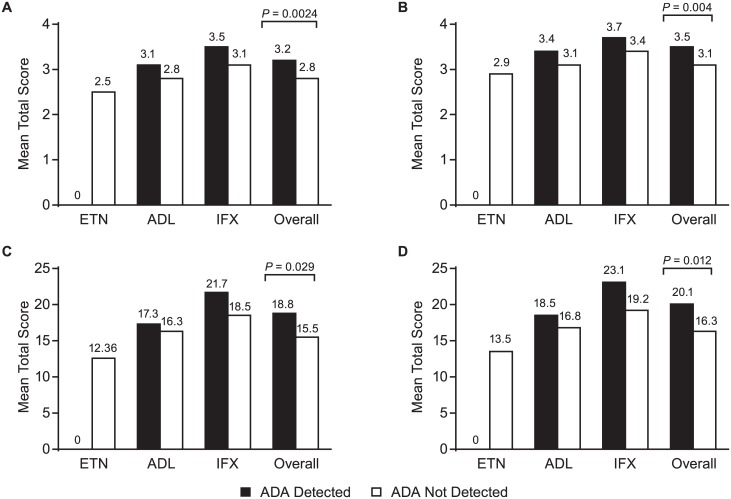
Relationship between ADA status and ESR (A, C, and E) or CRP (D-F) by treatment: ETN (A, D), ADL (B, E), IFX (C, F).

When data for all three TNF inhibitors were pooled, a total of 255/578 (44.1%) patients were in remission. A statistically significant (*P* = 0.0046) greater proportion of patients without detectable ADA (226/484; 46.7%) than those with detectable ADA (29/94; 30.9%) were in remission. Disease remission was observed in 106/197 (53.8%) patients treated with ETN (Fig 3C), 86/197 (43.7%) patients treated with ADL, and 63/184 (34.2%) patients treated with IFX. When patients treated with either ADL or IFX were considered individually, there was no difference in disease remission between patients with or without detectable ADA. Since there were no patients with detectable ADA among those treated with ETN, no correlations could be made with either ESR (Fig 4A) or CRP (Fig 4D). Among patients treated with ADL, there were significant differences between patients with and without the presence of detectable ADA and ESR (*P* = 0.0080; Fig 4B) and CRP (*P* = 0.0011; Fig 4E). Similar differences were observed for patients treated with IFX for ESR (*P* <0.0001; Fig 4C) and CRP (*P* = 0.0001; Fig 4F).

The mean (± SD) serum trough ETN concentration was 1.8 (1.03) μg/mL; the effect of ADA on serum trough drug concentration could not be determined for patients treated with ETN since no patient was positive for ADA. Among patients treated with ADL, the mean serum trough drug concentration was 80.5% lower in patients with detectable ADA (1.5 mg/mL) compared with those without detectable ADA (7.7 mg/mL; *P* < 0.0001). For patients treated with IFX, the mean serum trough drug concentration was 98.0% lower in patients with detectable ADA (0.2 mg/mL) compared with those without detectable ADA (9.8 mg/mL; *P* = 0.0003; Fig 3D).

**Fig 5:**
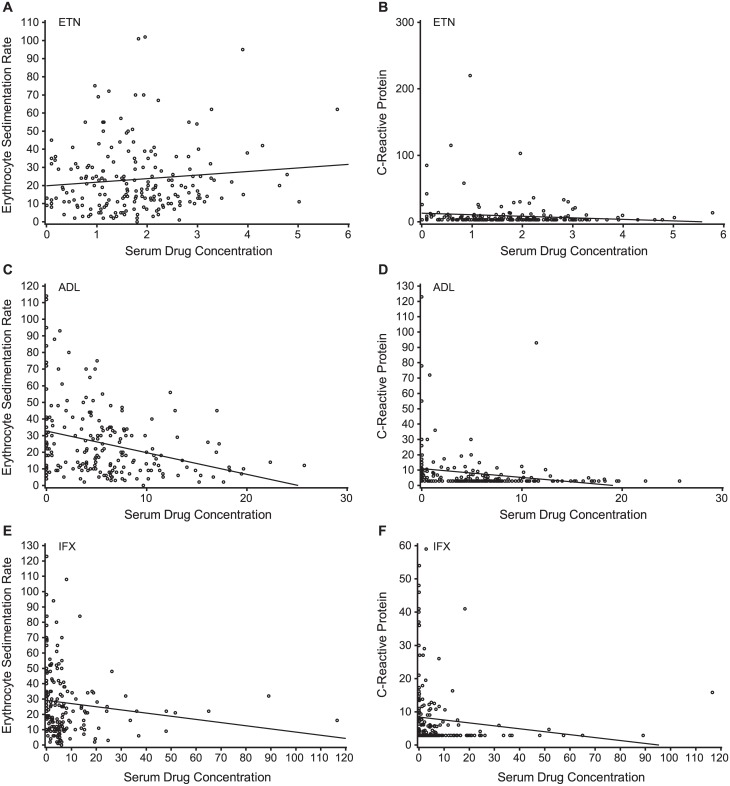
Mean total score by treatment and ADA status for (A) DAS28-ESR, (B) DAS28-CRP, (C) CDAI, and (D) SDAI. ADA = antidrug antibodies; DAS28 = disease activity score based on a 28-joint count; ESR = erythrocyte sedimentation rate; CRP = C-reactive protein; CDAI = clinical disease activity index; SDAI = simplified disease activity index; ETN = etanercept; ADL = adalimumab; IFX = infliximab.

Pooled data for all three TNF inhibitors showed differences for composite efficacy measures, DAS28-ESR (Fig 5A; *P* = 0.0024), DAS28-CRP (Fig 5B; *P* = 0.004), CDAI (Fig 5C; *P* = 0.029), and SDAI (Fig 5D; *P* = 0.012), all of which were higher in patients with detectable ADA compared with those without detectable ADA. However, there were no statistically significant differences between patients with and without detectable ADA within each treatment group.

**Fig 6:**
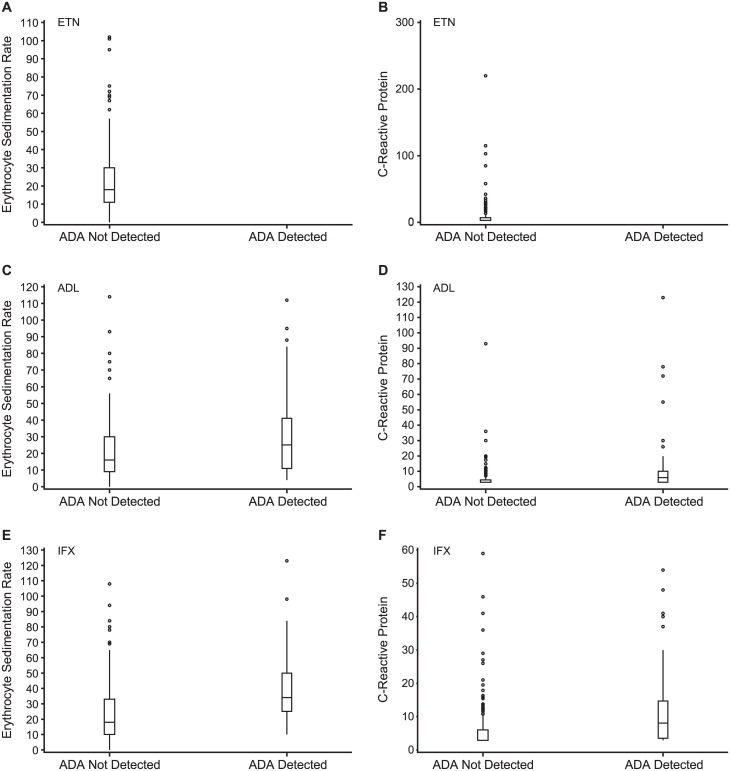
Correlation between serum trough drug concentration and ESR (A, C, and E) or CRP (B, D, and F) by treatment: ETN (A, B), ADL (C, D), IFX (E, F).

In patients treated with ADL or IFX, there were positive correlations between ADA titers and inflammation markers ESR (*P* = 0.0124 and 0.0001, respectively) and CRP (*P* < 0.0001 and = 0.0001, respectively; Table 2). The correlations with ADA titers for other efficacy endpoints assessed were low and not statistically significant. In patients treated with ETN, there were no significant correlations between serum trough drug concentration and efficacy endpoints assessed (Table 2) or inflammation markers, ESR (r = 0.073; *P* = 0.3074; Fig 5) and CRP (r = –0.139; *P* = 0.0505; Fig 6A and 6D). In patients treated with ADL, there were negative correlations between serum trough drug concentrations and DAS28-ESR (r = –0.172; *P* = 0.0155), DAS28-CRP (r = –0.154; *P* = 0.0297), 28-tender joint count (TJC; r = –0.167; *P* = 0.0187), ESR (r = –0.290; *P* < 0.0001; Fig 6B), and CRP (r = –0.440; *P* < 0.0001; Fig 6E). However, in patients treated with IFX, the only statistically significant negative correlations observed were between serum trough drug concentrations and ESR (r = –0.261; *P* = 0.0003; Fig 6C) and CRP (r = –0.399; *P* < 0.0001; Fig 6F).

**Table 2:** Correlations of efficacy measures with ADA titers and serum trough drug concentrations.

Parameter	ETN		ADL		IFX	
	CC	*P*-value	CC	*P*-value	CC	*P*-value
Correlation with ADA titers						
CDAI	NC		0.074	0.3008	0.053	0.4702
SDAI	NC		0.090	0.2124	0.068	0.3495
DAS28-ESR	NC		0.122	0.0916	0.090	0.2295
DAS28-CRP	NC		0.127	0.0766	0.084	0.2474
28-TJC	NC		0.103	0.1538	0.092	0.2049
28-SJC	NC		0.035	0.6223	0.032	0.6629
PGA	NC		0.076	0.2931	0.043	0.5524
PtGA	NC		0.077	0.2851	0.036	0.6237
ESR	NC		0.179	0.0124	0.288	0.0001
CRP	NC		0.382	< 0.0001	0.279	0.0001
Patient general health	NC		0.101	0.1593	0.024	0.7455
Correlation with serum trough drug concentration						
CDAI	–0.025	0.7224	–0.106	0.1351	0.044	0.5421
SDAI	–0.037	0.6032	–0.126	0.0765	0.017	0.8117
DAS28-ESR	–0.023	0.7521	–0.172	0.0155	0.029	0.6941
DAS28-CRP	–0.063	0.3790	–0.154	0.0297	0.017	0.8180
28-TJC	–0.100	0.1598	–0.167	0.0187	0.032	0.6534
28-SJC	0.001	0.9834	–0.025	0.7288	0.008	0.9083
PGA	0.035	0.6252	–0.128	0.0713	0.005	0.9400
PtGA	–0.024	0.7316	–0.065	0.3621	0.028	0.6979
ESR	0.073	0.3074	–0.290	<0.0001	–0.261	0.0003
CRP	–0.139	0.0505	–0.440	<0.0001	–0.399	<0.0001
Patient general health	0.025	0.7265	–0.081	0.2528	0.066	0.3585

ADA = anti-drug antibodies; ETN = etanercept; ADL = adalimumab; IFX = infliximab; CC = correlation coefficient; CDAI = Clinical Disease Activity Index; SDAI = Simplified Disease Activity Index; DAS28 = Disease Activity Score based on a 28-joint count; ESR = erythrocyte sedimentation rate; CRP = C-reactive protein; TJC = tender joint count; SJC = swollen joint count; PGA = physician global assessment; PtGA = patient global assessment.

### Patient-reported outcomes

In patients treated with ETN or IFX, no statistically significant correlations were observed between serum trough drug concentration and various patient-reported outcomes (Table 3). In patients treated with ADL, there was a statistically significant negative correlation between serum trough drug concentration and HAQ-DI (r = –0.225; *P* = 0.0014). In addition, there were statistically significant positive correlations between serum trough drug concentration and EQ-5D utility score (r = 0.177; *P* = 0.0126), EQ-5D visual analog scale (VAS) score (r = 0.224; *P* = 0.0014), SF-36 mental component score (r = 0.141; *P* = 0.0475), SF-36 Role Physical (r = 0.154; *P* = 0.0297), SF-36 General Health (r = 0.200; *P* = 0.0047), and SF-36 Role Emotional (r = 0.160; *P* = 0.0238).

**Table 3:** Correlations of serum trough drug concentration with PROs.

Parameter	ETN		ADL		IFX	
	CC	*P*-value	CC	*P*-value	CC	*P*-value
HAQ-DI	0.011	0.8791	–0.225	0.0014	–0.013	0.8568
EQ-5D Utility Score	0.030	0.6729	0.177	0.0126	0.001	0.9912
EQ-5D VAS Score	0.089	0.2118	0.224	0.0014	–0.057	0.4319
SF-36 (MCS)	0.063	0.3720	0.141	0.0475	0.026	0.7132
SF-36 (PCS)	–0.032	0.6544	0.138	0.0514	–0.070	0.3282
SF-36 (Physical Function)	–0.082	0.2467	0.129	0.0698	–0.074	0.3058
SF-36 (Role-Physical)	–0.027	0.7057	0.154	0.0297	0.015	0.8373
SF-36 (Bodily Pain)	0.076	0.2817	0.123	0.0826	–0.020	0.7861
SF-36 (General Health)	0.044	0.5405	0.200	0.0047	–0.065	0.3683
SF-36 (Vitality)	0.088	0.2133	0.108	0.1280	–0.069	0.3363
SF-36 (Social Functioning)	0.018	0.7955	0.117	0.1006	0.074	0.3071
SF-36 (Role-Emotional)	–0.060	0.4022	0.160	0.0238	–0.017	0.8148
SF-36 (Mental Health)	0.094	0.1862	0.114	0.1082	0.018	0.7985

PRO = patient-reported outcome; ADA = anti-drug antibodies; ETN = etanercept; ADL = adalimumab; IFX = infliximab; CC = correlation coefficient; HAQ-DI = Health Assessment Questionnaire-Disability Index; EQ-5D = EuroQol-5 Dimensions; VAS = visual analog scale; SF-36 = 36-Item Short-Form health survey; MCS = mental component score; PCS = physical component score.

### Safety

AEs were reported in a low percentage of patients in each treatment group (Table 4). AEs from infections and infestations were all mild or moderate in severity, except for one SAE of *C*. *difficile* colitis in the IFX treatment group. One SAE of renal failure was reported in the ETN treatment group, and one SAE of abnormal blood count was reported in the IFX treatment group; neither event was considered to be related to TNF inhibitor treatment. No malignancies were reported during the study. The incidence of four targeted medical events (injection site reaction, infusion reaction, serum sickness, and thromboembolic events) during the past 6 months on the current treatments was collected at the assessment visit. Injection site reactions were the most common AEs reported by patients treated with ETN (*n* = 17) and ADL (*n* = 10), whereas infusion reactions were the most common AE reported by patients treated with IFX (*n* = 4). In the IFX treatment group, serum sickness and a thromboembolic event were reported by one patient for each. No comparison could be made between patients with and without detectable ADA in the ETN-treated group since there were no patients with detectable ADA in this group. In ADL- and IFX-treated patients, there were no statistically significant differences in the proportion of patients reporting targeted medical history events between patients with and without detectable ADA.

**Table 4:** Summary of adverse events and targeted medical history.

	ETN (n = 200)		ADL (n = 199)		IFX (n = 196)*	
	ADA+ (n = 0)	ADA– (n = 200)	ADA+ (n = 62)	ADA– (n = 137)	ADA+ (n = 34)	ADA– (n = 161)
Patients with AEs, n (%)	5 (2.5)		4 (2.0)		12 (6.1)	
Patients with SAEs, n (%)	1 (0.5)		0		1 (0.5)	
Blood count abnormal, n (%)	0		0		1 (0.5%)	
Renal failure, n (%)	1 (0.5)		0		0	
Infections, n (%)	1 (0.5)		1 (0.5)		4 (2.0)	
Clostridium difficile colitis, n (%)	0		0		1 (0.5)	
Nasopharyngitis, n (%)	1 (0.5)		0		1 (0.5)	
Esophageal candidiasis, n (%)	0		0		1 (0.5)	
Respiratory tract infection, n (%)	0		1 (0.5)		0	
Upper respiratory tract infection, n (%)	0		0		1 (0.5)	
Targeted medical history^†^						
Injection site reactions, n (%)	0	17 (8.5)	1 (1.6)	9 (6.7)	0	0
Infusion reactions, n (%)	0	0	0	0	2 (5.9)	2 (1.2)
Serum sickness, n (%)	0	0	0	0	1 (2.9)	0
Thromboembolic events, n (%)	0	0	0	0	0	1 (0.6)

*One patient missing antibody results.

^†^Occurring while on the current biologic.

ETN = etanercept; ADL = adalimumab; IFX = infliximab; CC = correlation coefficient; ADA = anti-drug antibodies; AE = adverse event; SAE = serious adverse event.

## Discussion

To our knowledge, this was the first time that levels of ADA to ETN, ADL, and IFX were assessed in a single study using clinical samples from the same patient population, the same methodology, the same laboratory, and the same clinical data to evaluate information from a real-world clinical practice setting. This is important to ensure accurate and meaningful comparison of responses to the individual therapeutic agents. In this study, patients had to be on stable treatment for 6–24 months. This allowed sufficient time for patients to develop ADA to their treatment drug, thus enabling the evaluation of the effect of ADA on treatment response.

No patient treated with ETN (a soluble TNF receptor) had detectable ADA, whereas 24.4% of patients in the pooled ADL/IFX (anti-TNF monoclonal antibodies) group had detectable ADA; this was the pre-specified primary endpoint for this study. These data are consistent with previous reports indicating that ADL and IFX were more immunogenic than ETN [9–15,17–19]. The proportion of ADL-treated patients testing positive for ADA was consistent with previous reports (31% versus 28%) [12]. However, the observed proportion of IFX-treated patients testing positive for ADA (17%) was much lower than previous reports (up to 44%). Since the study was designed to include patients who were being treated with a TNF inhibitor for at least 6 months, patients who might have switched treatment due to the effects of ADA formation in the early phase of treatment were not included in the study population [12,20]. This interpretation is supported by our data showing a significantly higher incidence of ADA in patients treated with IFX for 6–12 months compared with those treated for 12–18 months or 18–24 months.

One challenge in understanding the true incidence of ADA is that samples with drug concentrations above the equimolar concentration of the ADA may test falsely negative due to interference by residual drug [21]. This study attempted to minimize the potential interference by collecting samples just prior to administration of the next dose when drug concentration would be expected to be lowest, i.e., trough drug levels, thereby providing the best opportunity to detect ADA. However, the collection of serum samples at the expected time of trough drug concentration may decrease the possibility of observing an association between drug concentration and clinical response, as drug concentrations at this time are likely to be low, both in patients who have a clinical response to treatment and in those who have not. However, in the absence of testing positive for ADA, trough concentrations are usually above the limit of quantification for most patients receiving the dosing regimens in this study.

In ETN-treated patients, the absence of any patients positive for ADA prevented comparisons between patients with and without detectable ADA in the proportion of patients with LDA, serum trough drug concentration, efficacy measures, health outcomes, or targeted medical history. Without the potential impact of ADA greatly lowering the trough concentrations of ETN, the range of observed concentrations was narrow. Consequently, there were also no statistically significant correlations of the serum trough drug concentration of ETN with efficacy measures or PROs.

In ADL- and IFX-treated patients, those who with detectable ADA had significantly lower serum trough drug concentrations and higher ESR and serum CRP levels, suggesting that the presence of ADA decreased the level of drug exposure in these patients and increased the markers for inflammation. Among patients treated with ADL or IFX when pooled or analyzed separately, there were no statistically significant differences observed between those with and without detectable ADA in the proportion of those with DAS28-ESR LDA. In addition, no statistically significant differences were observed between patients with and without detectable ADA in the proportion of those with DAS28-ESR remission, other efficacy measures, or HAQ-DI scores within each treatment group. This may have been due to the cross-sectional study design in which efficacy and health outcome analyses were based on a single assessment and not followed up over time.

In ADL-treated patients, there were statistically significant negative correlations of serum trough drug concentration with DAS28-CRP, DAS28-ESR, 28-TJC, ESR, and CRP concentration. Serum trough adalimumab concentration was also statistically significantly correlated with HAQ-DI, EQ-5D utility score, EQ-5D VAS score, and SF-36 general health score. In general, lower serum trough drug concentrations, potentially due to the presence of ADA, correlated with higher disease activity, suggesting reduced health-related quality of life and health status. In IFX-treated patients, serum trough drug concentration was statistically significantly negatively correlated with ESR and CRP concentration, but was not statistically significantly correlated with efficacy measures or health outcome measures assessed. Although the current study did show lower trough concentrations for ADL and IFX in patients testing positive for ADA, with the exception of ESR and CRP values there were no within-group differences in disease activity between patients with and without detectable ADA.

The lack of a relationship between ADA status and clinical responses/health outcomes overall may have been due to a limitation of the cross-sectional design of this study. Only patients who were still responding reasonably well to a given treatment drug and thus still receiving it were recruited. Patients with higher disease activity who may have already discontinued that treatment drug were not eligible for this study. Furthermore, the use of other conventional treatments for RA was not restricted in this study. The question of the effect of ADA status on clinical and health outcomes responses may be better addressed by a prospective study where the rate of discontinuation of treatment could be assessed among patients with and without detectable ADA over time.

All three TNF inhibitors were well tolerated, with AEs and SAEs comparable to previous reports. A targeted medical history of injection site reactions, infusion reactions, serum sickness, and thromboembolic events reported while on current anti-TNF therapy was not statistically significantly different between patients with and without detectable ADA. This could very well be because of the small number of such events reported in this study, which did not allow for an adequate statistical comparison. Overall, the AE profile was as expected in a population of patients with RA receiving treatment with a TNF inhibitor.

Although the cross-sectional design of this study provided a very useful snap shot of every day experiences of physicians in clinical practice, it also revealed some limitations that affected the interpretation of the results. Since data were collected at only a single time point without follow-up, no trends on how patients responded over time nor the impact of historical treatment could be evaluated. In addition, since patients had to be receiving at least 6 months of continuous treatment with the same agent to be eligible for this study, there is an inherent bias towards those patients who responded well to their treatment. Consequently, the data reported in this paper are limited to only these patients. Patients who did not respond well to any of the three agents being evaluated may have been excluded by the study design, and as such, the incidence and impact of ADA in these patients could not be evaluated. Furthermore, since there were no restrictions on concomitant or prior treatments, the impact of these treatments either individually or collectively on the development of ADA, clinical responses to treatment, or health outcomes could not be evaluated. A total of 9 different prior biologic treatments were recorded. In addition, there was also a group of blinded investigational drugs which were reported as biologic. A variety of other reasons for prior treatment discontinuation including payer/insurance, drug intolerance, and conclusion of prior trial participation were also reported. Consequently, the number of prior biologics should not be assumed as failure of those treatments. Finally, the possibility of these data being a result of chance cannot be definitively ruled out due to a lack of statistically significant differences in clinical outcomes between patients with and without detectable ADA.

In conclusion, these results indicated that in this relatively large, multinational, cross-sectional real-world population of patients with RA receiving treatment for 6 to 24 months, ADA developed in a higher proportion of patients receiving an anti-TNF monoclonal antibody (ADL or IFX) than in patients receiving a soluble dimeric TNF receptor fusion protein (ETN). The presence of antibodies to ADL or IFX was associated with corresponding lower serum drug concentrations and efficacy outcomes. All three agents were well tolerated, with no significant differences between patients with and without detectable ADA. In addition, there was some indication in the ADL- and IFX-treated patients that decreased drug concentration due to the presence of ADA may lead to a reduction in health outcome measures. A longitudinal study design may be necessary to further investigate the impact of ADA on the response to TNF inhibitor treatment in patients with RA.

## Supporting information

S1 Checklist trendstatement_trend_checklist_Completed.pdf. (PDF)

S1 File B1801364 ANTIBODY-RA_Protocol 05SEP2013_final PDF.pdf. (PDF)
