# Correction: The impact of anti-drug antibodies on drug concentrations and clinical outcomes in rheumatoid arthritis patients treated with adalimumab, etanercept, or infliximab: Results from a multinational, real-world clinical practice, non-interventional study

**DOI:** 10.1371/journal.pone.0179308

**Published:** 2017-06-05

**Authors:** Robert J. Moots, Ricardo M. Xavier, Chi Chiu Mok, Mahboob U. Rahman, Wen-Chan Tsai, Mustafa H. Al-Maini, Karel Pavelka, Ehab Mahgoub, Sameer Kotak, Joan Korth-Bradley, Ron Pedersen, Linda Mele, Qi Shen, Bonnie Vlahos

The images for Figs 4, 5, and 6 are incorrectly switched. The image that appears as Fig 4 should be Fig 5, the image that appears as Fig 5 should be Fig 6, and the image that appears as Fig 6 should be Fig 4. The figure captions appear in the correct order.
