# Supplementary material for: The impact of anti-drug antibodies on drug concentrations and clinical outcomes in rheumatoid arthritis patients treated with adalimumab, etanercept, or infliximab: Results from a multinational, real-world clinical practice, non-interventional study
Source: PLoS One. 2017 Apr 27;12(4):e0175207. doi: 10.1371/journal.pone.0175207 (PMC5407581; doi:10.1371/journal.pone.0175207)
Supplement: S1 File — (PDF) [file pone.0175207.s002.pdf]

NON-INTERVENTIONAL (NI) STUDY PROTOCOL

Study information

**Title**	Anti-TNF Inhibitor Antibody-Mediated Blockade of Drug Efficacy in Rheumatoid Arthritis (ANTIBODY-RA)
**Protocol number**	B1801364
**Protocol version identifier**	Original Protocol
**Date of latest version of protocol**	05 Sep 2013
**Active substance**	Etanercept (Enbrel)
**Medicinal product**	Etanercept (Enbrel)
**Product reference**	EU/1/99/126/001-022
**Procedure number**	EMEA/H/C/262

This document contains confidential information belonging to Pfizer. Except as otherwise agreed to in writing, by accepting or reviewing this document, you agree to hold this information in confidence and not copy or disclose it to others (except where required by applicable law) or use it for unauthorized purposes. In the event of any actual or suspected breach of this obligation, Pfizer must be promptly notified.

090177e184841661\Approved\Approved On: 09-Sep-2013 19:01

SCHEDULE OF ACTIVITIES

The Schedule of Activities table provides an overview of the study visits and assessments. Refer to Study Procedures (Section 5.2.3) and Assessments (Section 5.3) for detailed information on assessments evaluated in the study.

Study Activity	Screening[a]	Assessment Visit[b]	Follow-Up[c]
Informed consent	X		
Current biological treatment/dosing date	X	X	
Demographics	X		
Date of RA diagnosis	X		
Review eligibility criteria	X	X	
Screen and enroll in IRT	X	X	
Prior/concomitant RA medications		X	
Joint Assessment (28 Count) [d, e]		X	
Health Assessment Questionnaire- Disability Index (HAQ-DI)		X	
Subject General Health VAS[d]		X	
Physician Global Assessment of Arthritis VAS[e]		X	
Subject Global Assessment of Arthritis VAS[e]		X	
36-Item Short-Form Health Survey (SF-36)		X	
EuroQol-5 Dimensions (EQ-5D)		X	
Erythrocyte sedimentation rate (ESR)[d,e]		X	
C-Reactive protein (CRP)[d,e]		X	
Etanercept, adalimumab, or infliximab trough drug concentration measurements and corresponding antidrug antibody testing		X	
Targeted medical history[f]		X	
Adverse event monitoring[g]	X	X	X
End of Study Subject Summary			X

a. Screening and assessment visit procedures may be combined into one visit, provided that visit occurs within 2 days prior to or on the date of the next scheduled dose (before dosing) of etanercept, adalimumab, or infliximab, as applicable. If the patient is not within this period relative to next scheduled dose, the assessment data may be collected at a subsequent visit occurring when the patient is at their trough concentration.

b. Collection of assessment data is to occur at the patient's trough drug concentration which is defined as the period within 2 days prior to or on the date of the next scheduled dose (before dosing) of etanercept, adalimumab, or infliximab, as applicable. Assessment data must be collected ≤3 months from the screening date. If a subsequent routine visit when the patient is at trough level does not occur within three months of the screening visit, the patient will not be enrolled and no data will be collected. If the assessment visit is on the day of the next scheduled dose, all data should be collected prior to administration of the next scheduled dose.

c. The follow up visit consists of a telephone call to assess new adverse events. It should occur approximately 28 days following the assessment visit.

090177e184841661\Approved\Approved On: 09-Sep-2013 19:01

d. These procedures constitute the Disease Activity Score (DAS28).

e. These procedures constitute the Clinical Disease Activity Index (CDAI) and Simplified Disease Activity Index (SDAI) Scores. CDAI score does not include ESR or CRP.

f. Targeted medical history includes injection site reactions, infusion reactions, serum sickness, and thromboembolic events.

g. For non-serious and serious adverse events, the reporting period to Pfizer or its designated representative begins from the time of the patient's first dosing in the observational period as per study design through and including 28 calendar days after the last administration of the study drug within the observational period. The observational period in this study starts after subject signing of informed consent and ends at the time of collection of all data outlined at the assessment visit.

090177e184841661\Approved\Approved On: 09-Sep-2013 19:01

TABLE OF CONTENTS

LIST OF FIGURES 6
1. LIST OF ABBREVIATIONS..7
2. DOCUMENT HISTORY: AMENDMENTS AND UPDATES 9
3. RATIONALE AND BACKGROUND..10
4. RESEARCH QUESTION AND OBJECTIVES 10
4.1. Objectives...10
4.2. Endpoints..11
4.2.1. Primary Endpoint..11
4.2.2. Secondary Endpoints 11
4.2.3. Exploratory Endpoints...11
5. RESEARCH METHODS 12
5.1. Study Design 12
5.2. Setting...13
5.2.1. Inclusion Criteria 13
5.2.2. Exclusion Criteria 14
5.2.3. Study Procedures 14
5.2.3.1. Screening...14
5.2.3.2. Assessment Visit 15
5.2.3.3. Follow-up Telephone Contact..16
5.3. Assessments 16
5.3.1. Efficacy Assessments 17
5.3.1.1. Clinical Laboratory Evaluations...17
5.3.1.2. DAS28 Assessments 17
5.3.1.3. Joint Assessment of Tender/Painful Joint Count and Swollen Joint Count (28)...17
5.3.1.4. Simplified Disease Activity Index (SDAI) and Clinical Disease Activity Index (CDAI) 17
5.3.1.5. Physician's Global Assessment (PGA) 17
5.3.1.6. Subject's Global Assessment 18
5.3.1.7. Subject's General Health VAS...18
5.3.2. Health Outcome Assessments...18

090177e184841661\Approved\Approved On: 09-Sep-2013 19:01

5.3.2.1. Health Assessment Questionnaire (Disability and Discomfort Scales) (HAQ-DI) 18
5.3.2.2. Euro Qol EQ-5 Dimensions Questionnaire (EQ-5D)................19
5.3.2.3. Short Form-36 Health Survey (SF-36)....................................19
5.3.3. Safety Assessments...19
5.3.3.1. Targeted Medical History...19
5.3.4. Drug Concentration and Antidrug Antibody Assessments.........................19
5.3.4.1. Serum for Analysis of Etanercept, Adalimumab, and Infliximab 19
5.3.4.2. Serum for Analysis of Immunogenicity: Etanercept, Adalimumab, and Infliximab Antidrug Antibody 20
5.4. Study Size..20
5.5. Data Management 20
5.5.1. Case Report Forms/Electronic Data Record...20
5.5.2. Record Retention 21
5.6. Data Analysis 21
5.7. Quality Control..22
6. PROTECTION OF HUMAN SUBJECTS 23
6.1. Patient Information and Consent...23
6.2. Patient Withdrawal..23
6.3. Institutional Review Board (IRB)/Independent Ethics Committee (IEC) 23
6.4. Ethical Conduct of the Study 23
7. ADVERSE EVENT REPORTING...24
7.1. Adverse Events...24
7.2. Reporting Period 24
7.3. Definition of Adverse Event 24
7.4. Abnormal Test Findings...25
7.5. Serious Adverse Events..26
7.6. Hospitalization 26
7.7. Causality Assessment..27
7.8. Exposure During Pregnancy...27
7.9. Medication Error 28
7.10. Reporting Requirements..29

090177e184841661\Approved\Approved On: 09-Sep-2013 19:01

7.11. Serious or Non-Serious Adverse Event Reporting Requirements........................29
7.12. Single Reference Safety Document...30
8. PLANS FOR DISSEMINATING AND COMMUNICATING STUDY RESULTS..........30
8.1. Communication of Results by Pfizer 30
8.2. Publications by Investigators 30
9. REFERENCES 32
10. LIST OF FIGURES 33
11. APPENDICES 34

LIST OF FIGURES

Figure 1. Study Schematic 13

APPENDICES

Appendix 1. 1987 Revised Criteria for the Classification of Rheumatoid Arthritis................34
Appendix 2. Sample Health Assessment Questionnaire – Disability Index 35
Appendix 3. Sample EQ-5D Questionnaire..37
Appendix 4. Sample 36-Item Short-Form Health Survey 40

090177e184841661\Approved\Approved On: 09-Sep-2013 19:01

1. LIST OF ABBREVIATIONS

Abbreviation	Definition
ACR	American College of Rheumatology
CDAI	Clinical Disease Activity Index
CRF	Case report form
CRP	C-reactive protein
DAS28	Disease Activity Score based on a 28-joint count
DMARD	Disease modifying antirheumatic drug
DVT	Deep vein thrombosis
ELISA	Enzyme-linked immunosorbent assay
EQ-5D	Euro Qol EQ-5 Dimensions Questionnaire
ESR	Erythrocyte sedimentation rate
FDA	US Food and Drug Administration
FDAAA	US Food and Drug Administration Amendments Act of 2007
GH	Subject General Health Visual Analog Scale
HAQ-DI	Health Assessment Questionnaire-Disability Index
IEC	Independent Ethics Committee
IgG_1	human immunoglobulin G_1
IRB	Institutional Review Board
IRT	Interactive response technology (patient enrollment tracking)
LDA	Low disease activity
LSLV	Last Subject Last Visit
mAB	Monoclonal antibody

090177e184841661\Approved\Approved On: 09-Sep-2013 19:01

MCP	Metacarpophalangeal joints proximal
MI	Myocardial infarction
MTX	Methotrexate
NSAID	Nonsteroidal anti-inflammatory drug
PCD	Primary outcome completion date
PIP	Proximal interphalangeal joints
RA	Rheumatoid arthritis
RIA	Radioimmunoassay
SAP	Statistical Analysis Plan
SDAI	Simplified Disease Activity Index
SF-36	36-Item Short-Form Health Survey
TNF	Tumor necrosis factor
VAS	Visual analog scale

090177e184841661\Approved\Approved On: 09-Sep-2013 19:01

2. DOCUMENT HISTORY: AMENDMENTS AND UPDATES

Original Protocol Date: 05 Sep 2013

Amendment number	Date	Substantial or administrative amendment	Protocol section(s) changed	Summary of amendment(s)	Reason

090177e184841661\Approved\Approved On: 09-Sep-2013 19:01

3. RATIONALE AND BACKGROUND

Rheumatoid arthritis (RA) is a systemic disease characterized by a chronic and persistent inflammation and juxta-articular bone destruction. Treatment using tumor necrosis factor (TNF) inhibitors such as etanercept, infliximab, and adalimumab has significantly improved the outcome in patients with RA that have not responded to conventional disease-modifying antirheumatic drug (DMARD) therapy.

Etanercept is a soluble TNF receptor fusion protein, structurally different from the anti-TNF monoclonal antibodies (mAB) adalimumab, and infliximab. It is a human, soluble, dimeric, TNF type II receptor, TNFR-p75 (formerly called TNFR-p80), that is linked to human immunoglobulin G_1 (IgG_1)-Fc moiety. Infliximab is a mouse-human chimeric mAB against TNF. Adalimumab is a third TNF inhibitor which is a fully human mAB.[1]

The administration of any biological therapeutic has the potential of eliciting an unwanted immune response, culminating in the development of antidrug antibodies, which may pose problems for both patient's safety as well as drug efficacy.[2] The immunogenic potential of a molecule is dependent upon a variety of factors related to the product. Emerging data has suggested the association between the development of antidrug antibodies and diminished drug concentration and treatment response after an initial good response.[3,4] Antidrug antibodies may also contribute to the development of safety concerns such as injection site and infusion reactions, thromboembolic events, and serum sickness.[3,5,6]

Unlike infliximab and adalimumab which have reported incidences of development of antidrug antibodies of 42%[7] and 28%[8] respectively, etanercept treatment has been demonstrated to induce a significantly lower immunogenic response and has not been associated with the development of detectable neutralizing antibodies with continued treatment.[9,10] Currently there is a lack of comparative data to illustrate the occurrence of antidrug antibody for these three leading biologic treatments for rheumatoid arthritis and their association with drug concentration, clinical efficacy, as well as targeted medical history since the data are from different studies utilizing different design and assay methodologies.

The purpose of this study is to assess immunogenicity, serum trough drug concentration, and efficacy for all three biologic anti-TNF therapies in a cross-sectional population of RA patients in a consistent, controlled manner.

4. RESEARCH QUESTION AND OBJECTIVES

4.1. Objectives

The primary objective of this study is to evaluate the proportion of patients positive for antidrug antibodies treated with a soluble receptor TNF inhibitor (etanercept) versus patients positive for antidrug antibodies treated with monoclonal antibody TNF inhibitors (adalimumab and infliximab).

090177e184841661\Approved\Approved On: 09-Sep-2013 19:01

The secondary objective is to evaluate the relationship between immunogenicity, serum drug concentration, and efficacy/health outcomes of patients treated with etanercept, adalimumab, or infliximab.

4.2. Endpoints

4.2.1. Primary Endpoint

Proportion of patients positive for antidrug antibodies among those treated with etanercept versus those treated with monoclonal antibodies (adalimumab or infliximab).

4.2.2. Secondary Endpoints

Secondary endpoints will be analyzed for the groups of patients currently treated with each of the anti-TNF therapies, and also for the overall combined sample, where applicable (see Section 5.6 Data Analysis). Efficacy measures include the CDAI, SDAI, DAS28-ESR and DAS28-CRP scores, and health outcomes measures include the HAQ-DI, SF-36 and EQ-5D (see Sections 5.3.1 Efficacy Assessments and 5.3.2 Health Outcome Assessments).

1. Proportion of patients with Low Disease Activity (LDA) (DAS28-ESR score ≤3.2) among those who are antidrug antibody positive versus negative (all patients receiving etanercept, adalimumab, or infliximab combined).
2. Serum trough drug concentrations for etanercept, adalimumab, and infliximab compared between patients who are antidrug antibody positive versus negative.
3. Proportion of patients positive for antidrug antibodies among those treated with etanercept, adalimumab, or infliximab.
4. Efficacy measures compared between patients who are antidrug antibody positive versus negative.
5. HAQ-DI scores compared between patients who are antidrug antibody positive versus negative.
6. Correlation of antidrug antibody titers with efficacy measures.
7. Correlation of antidrug antibody titers with trough drug concentration.

4.2.3. Exploratory Endpoints

Exploratory endpoints will be analyzed for the groups of patients currently treated with each of the anti-TNF therapies, and also for the overall combined sample, where applicable (see Section 5.6 Data Analysis).

1. Individual components of efficacy measures compared between patients who are antidrug antibody positive versus negative.

090177e184841661\Approved\Approved On: 09-Sep-2013 19:01

2. Correlation of antidrug antibody titers with individual components of efficacy measures.

3. Correlation of serum trough drug concentration with total scores and individual components of efficacy measures.

4. Proportion of patients with targeted medical history (injection site reactions, infusion reactions, serum sickness, and thromboembolic events), occurring while on current anti-TNF treatment, among patients who are antidrug antibody positive versus negative.

5. Proportion of patients positive for antidrug antibodies among those who are concurrently treated with methotrexate (MTX) versus not concurrently treated with MTX.

6. Quantification of dose and serum trough drug concentration in the 4 subsets of patients determined by antibody status (positive/negative) and clinical response (LDA/nonLDA).

7. Health outcomes measures compared between patients who are antidrug antibody positive versus negative.

8. Correlation of serum trough drug concentration with health outcomes measures.

5. RESEARCH METHODS

5.1. Study Design

This multicenter, non-interventional study will evaluate data collected from a cross-sectional population of patients with rheumatoid arthritis who are currently receiving treatment with either etanercept, adalimumab, or infliximab for a minimum of 6 months and maximum of 24 months prior to the study assessment visit.

Approximately 600 patients treated in a clinical setting with one of the three targeted therapies will be enrolled. The number of patients on each therapy will be monitored using interactive response technology (IRT) to assure data for approximately 200 patients is included for each RA treatment. Inclusion in each group will be competitive and once 200 patients for a treatment have been enrolled, that group will be closed and no further data will be collected. Data and laboratory samples will be collected in a single study visit for the assessment of treatment history and dosing, disease status, patient-reported outcomes, serum trough drug concentration, antidrug antibody status, and targeted medical history (injection site reactions, infusion reactions, serum sickness, and thromboembolic events).

Results from antidrug antibody assays will be applied towards analysis of the primary endpoint: proportion of patients positive for antidrug antibodies among patients treated with etanercept versus patients treated with adalimumab or infliximab, combined. Secondary endpoints numbered 1 and 2 will be analyzed through comparison of patients with low disease activity, as defined by a DAS28 (ESR) score of ≤3.2, between antidrug antibody

090177e184841661\Approved\Approved On: 09-Sep-2013 19:01

positive and negative patients (all treatments combined), and the comparison of serum trough drug concentrations in antibody positive versus antibody negative patients for etanercept, adalimumab, and infliximab. Other secondary efficacy and exploratory endpoints are described in Sections 4.2.2 and 4.2.3, respectively, of this document.

5.2. Setting

This study is designed to evaluate data collected from a cross-sectional population of patients treated with anti-TNF therapies, etanercept, adalimumab, and infliximab. Therefore, investigators should consider all potentially eligible RA patients consecutively seen at the clinic who are currently treated with etanercept, adalimumab, or infliximab for the appropriate prestudy duration (a minimum of 6 months up to a maximum of 24 months) for participation in this study. No criteria other than those outlined in this protocol should be applied towards selection of suitable patients, including disease status, patient/investigator satisfaction with current treatment, concomitant therapies, or other considerations.

The observational period for this study is defined as the period of time from signing of informed consent through and including the collection of all data outlined at the assessment visit. If the assessment visit does not occur within 3 months of the date of screening, the patient will not be enrolled and no assessment data will be collected.

Figure 1. Study Schematic

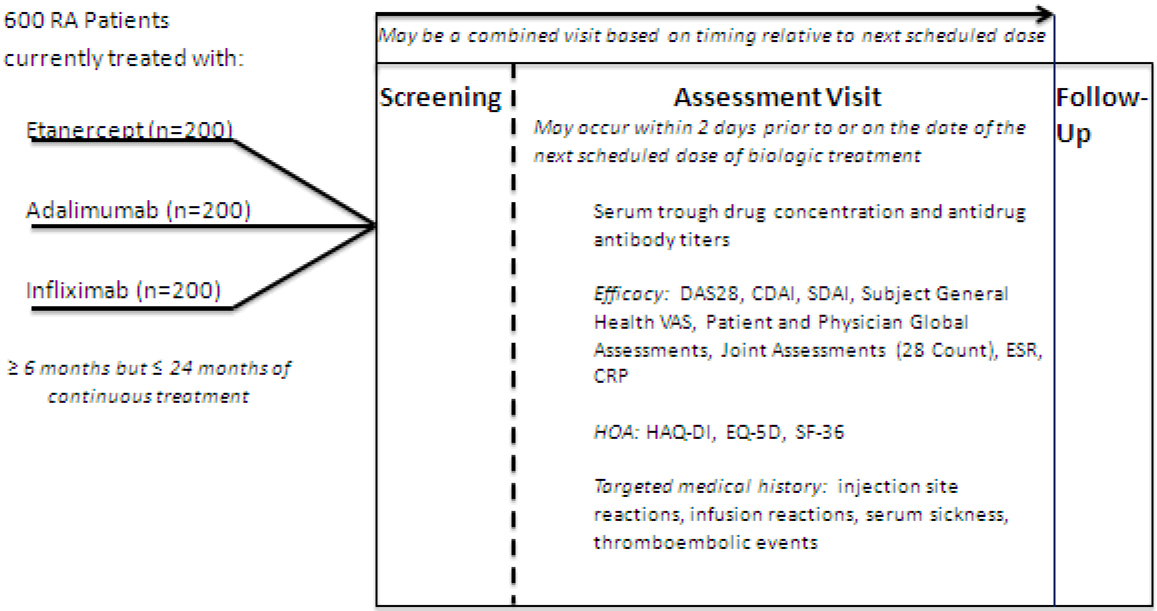

5.2.1. Inclusion Criteria

Patients must meet all of the following inclusion criteria to be eligible for inclusion in the study:

1. Age ≥18 years.

090177e184841661\Approved\Approved On: 09-Sep-2013 19:01

2. Diagnosis of RA based on the 1987 American College of Rheumatology (ACR) revised criteria (Appendix 1).

3. Current continuous treatment with either etanercept, adalimumab, or infliximab for a minimum of 6 months and maximum of 24 months prior to the study assessment visit.

4. Evidence of a personally signed and dated informed consent document indicating that the patient (or a legally acceptable representative) has been informed of all pertinent aspects of the study.

5.2.2. Exclusion Criteria

Patients meeting any of the following criteria will not be included in the study:

1. Treatment with biosimilar or investigational etanercept, adalimumab, or infliximab within 6 months of the assessment study visit.

2. Treatment with any other investigational drug within 3 months or five half-lives of the drug, whichever is longer, of the assessment study visit.

3. Any medical condition that would interfere with efficacy or other assessments (eg, fibromyalgia, lupus).

4. Difficulty reading or understanding local language.

5.2.3. Study Procedures

Screening and collection of data may be performed at one visit following signing of informed consent and eligibility evaluation. If a patient's screening visit does not occur at a time when they are at trough drug concentration (within 2 days prior to or on the date of their next scheduled dose, before dosing), the assessment data may be collected at a subsequent visit occurring when the patient is at their trough concentration.

If a subsequent routine visit when the patient is at trough level does not occur within three months of the screening visit, the patient will not be enrolled and no data will be collected.

Post enrollment data collection on efficacy measures, health outcomes, and blood tests will be performed in the course of a single visit. The study blood samples will be collected in conjunction with routine blood sample collection associated with patient care before any drug administration to obtain the trough drug concentration measurement.

5.2.3.1. Screening

Screening procedures should not commence until after the informed consent form for this study has been signed. Patients declared as screen failures due to insufficient duration of treatment with etanercept, adalimumab, or infliximab or for exclusionary criteria surrounding prior biologic or investigational treatments may be considered for re-screening should they become eligible in the future. The following information will be collected at the screening visit:

090177e184841661\Approved\Approved On: 09-Sep-2013 19:01

- Sign and date the informed consent.
- Review inclusion and exclusion criteria.
- Document current biologic treatment for RA, either etanercept, adalimumab, or infliximab, including current dose, frequency and date of last dose. Determine next scheduled dosing date.
- Record patient demography (including date of birth, gender, race, ethnicity, and date of RA diagnosis).
- Register eligible patient as "screened" in the IRT system, otherwise register as a screen failure.
- Record AEs on the appropriate CRF (see Section 7.1 Adverse Events and Section 7.2 Reporting Period).

If the patient meets criteria for enrollment and is within 2 days prior to or on the date of their next scheduled dose of etanercept, adalimumab, or infliximab, then assessment visit procedures may be performed on the same day.

5.2.3.2. Assessment Visit

Patients determined eligible for inclusion in the study and whose next scheduled dose of etanercept, adalimumab, or infliximab is within two days of the screening evaluation may have data collected for the assessment visit on the same day as screening. In this case, data collected at screening does not need to be collected again on the assessment visit case report forms (CRFs) (as indicated below). If the assessment visit is performed on the day of the patient's next scheduled dose, all study data should be collected prior to administration of that dose.

All assessment procedures should be completed on the same day. The following data will be collected at the assessment visit:

- Review inclusion and exclusion criteria (unless screening and assessment visit are performed on the same day).
- Register eligible patient as "enrolled" in the IRT system, otherwise register as a screen failure.
- Review dose, frequency and date of last dose of the current biologic treatment for RA (etanercept, adalimumab, or infliximab), and revise if applicable (unless screening and assessment visit are performed on the same day).
- Record additional prior and concomitant medications used for the treatment of RA, including methotrexate, other DMARDs, corticosteroids, NSAIDs, and any other prior biologic therapies.

090177e184841661\Approved\Approved On: 09-Sep-2013 19:01

- Record any targeted medical history of injection site reactions, infusion reactions, serum sickness, and thromboembolic events.
- Collect samples for the following laboratory evaluations to be shipped to the bioanalytical laboratory:
  - C-Reactive protein (CRP);
  - Etanercept, adalimumab, or infliximab drug concentration and corresponding antidrug antibody titers.
- Collect sample and perform erythrocyte sedimentation rate (ESR) testing.
- Assessment of Tender/Swollen Joints (28 count).
- Physician Global Assessment of Arthritis Visual Analog Scale (VAS).
- Subject Global Assessment of Arthritis VAS.
- Subject General Health VAS
- Health Assessment Questionnaire - Disability Index (HAQ-DI).
- EuroQol-5 Dimensions (EQ-5D).
- 36-Item Short-Form Health Survey (SF-36).
- Record AEs on the appropriate CRF (see Section 7.1 Adverse Events and Section 7.2 Reporting Period).

5.2.3.3. Follow-up Telephone Contact

Approximately 28 days after the assessment visit there should be a follow-up telephone call to assess new and ongoing AEs, as appropriate.

- Record AEs on the appropriate CRF (see Section 7.1 Adverse Events and Section 7.2 Reporting Period).
- Complete the end of study subject summary CRF.

5.3. Assessments

Every effort should be made to ensure that data is collected on the assessments as outlined by the protocol.

090177e184841661\Approved\Approved On: 09-Sep-2013 19:01

5.3.1. Efficacy Assessments

5.3.1.1. Clinical Laboratory Evaluations

Samples for CRP evaluation will be collected and sent to the bioanalytical laboratory. ESR will be performed locally.

5.3.1.2. DAS28 Assessments

The DAS28 assessment is a derived measurement with differential weight given to each component[11] DAS28 will be calculated twice, utilizing first ESR, and then CRP as the acute phase reactant:

- DAS28-ESR = 0.56 sqrt (28 painful/tender joint count) + 0.28 sqrt (28 swollen joint count) + 0.70 (ln ESR) + 0.014 GH, where GH=subject general health VAS (0-100 mm).

- DAS28-4 CRP = 0.56 sqrt (28 painful/tender joint count) + 0.28 sqrt (28 swollen count) + 0.36 (ln CRP+1) + 0.014 GH + 0.96, where GH=subject general health VAS (0-100 mm).

The specific components of the DAS28 assessment that will be used in this study are: Tender/Painful Joint Count (28), Swollen Joint Count (28), ESR/CRP, and Subject's General Health VAS assessment.

5.3.1.3. Joint Assessment of Tender/Painful Joint Count and Swollen Joint Count (28)

The 28 tender/painful joint count and swollen joint count includes the following joints: shoulders, elbows, wrists, metacarpophalangeal joints (MCP), proximal interphalangeal joints (PIP), and knees.

5.3.1.4. Simplified Disease Activity Index (SDAI) and Clinical Disease Activity Index (CDAI)

The SDAI and CDAI[12] are defined as:

- SDAI = DAS 28 prorated Swollen Joint Count (0-28) + DAS 28 prorated Tender Joint Count (0-28) + Physician's Global Assessment (0-10) + Subject's Global Assessment (0-10) + CRP (in mg/dL).

- CDAI = DAS 28 prorated Swollen Joint Count (0-28) + DAS 28 prorated Tender Joint Count (0-28) + Physician's Global Assessment (0-10) + Subject's Global Assessment (0-10).

5.3.1.5. Physician's Global Assessment (PGA)

The investigator will estimate the patient's overall disease activity over the last 2-3 days (this should be independent of the Subject's Global Assessment) using a scale between 0 (no disease activity) and 10 (extreme disease activity). One number is to be marked with 'X'.

090177e184841661\Approved\Approved On: 09-Sep-2013 19:01

The following is a sample representation of the questionnaire:

No disease activity over last 2-3 days												Extreme Disease Activity over last 2-3 days
	0	1	2	3	4	5	6	7	8	9	10	

5.3.1.6. Subject's Global Assessment

Patients will assess their overall disease activity over the last 2-3 days using a scale between 0 (no disease activity) and 10 (extreme disease activity), which corresponds to the magnitude of their pain). One number is to be marked with 'X'.

The following is a sample representation of the questionnaire.

No disease activity over last 2-3 days												Extreme Disease Activity over last 2-3 days
	0	1	2	3	4	5	6	7	8	9	10	

5.3.1.7. Subject's General Health VAS

Patients are asked to answer the question "In general how would you rate your health over the last 2-3 weeks?" by marking the 100 mm visual analog scale with a single slash mark that intersects with the line. The length of the line is measured from the left (in mm). Note: the VAS line on the questionnaire will measure 100 mm.

The following is a sample representation of the questionnaire.

Very Well |————————————————| Extremely Bad
0 mm 100 mm

5.3.2. Health Outcome Assessments

The health outcomes assessments have been included in this study to explore the patient's own perceptions about his or her level of disease activity and health-related quality of life.

Patients will complete all questionnaires using pen and paper. These questionnaires should be completed prior to any procedures being performed at the visit, if possible. Once the patient has completed the forms, the site staff should check them for completeness before the patient leaves the clinic.

Every effort should be made to obtain unbiased responses from the patient on these questionnaires. Site staff and the investigator must not influence the patient's responses.

5.3.2.1. Health Assessment Questionnaire (Disability and Discomfort Scales) (HAQ-DI)

The HAQ-DI assesses the degree of difficulty a patient has experienced during the past week in 8 domains of daily living activities: dressing and grooming, arising, eating, walking, hygiene, reach, grip, and other activities. Each activity category consists of 2-3 items. For each question in the questionnaire, the level of difficulty is scored from 0 to 3 with 0 representing "no difficulty," 1 as "some difficulty," 2 as "much difficulty," and 3 as

090177e184841661\Approved\Approved On: 09-Sep-2013 19:01

"unable to do." Any activity that requires assistance from another individual or requires the use of an assistive device adjusts to a minimum score of 2 to represent a more limited functional status. A copy of the questionnaire can be found in Appendix 2.

5.3.2.2. Euro Qol EQ-5 Dimensions Questionnaire (EQ-5D)

The EQ-5D is a patient-completed questionnaire designed to assess health related quality of life. There are two components to the EuroQol (EQ-5D): a Health State Profile and a VAS. For the Health State Profile, patients record their level of current health for five domains comprising a health profile: mobility, self-care, usual activities, pain/discomfort, and anxiety/depression. Scores from the five domains may be used to calculate a single index value, also known as a utility score. On the VAS patients are asked to rate their current health on a scale from 0 to 100 -mm scale where 0 represents the 'worst imaginable health state' and 100 represents the 'best imaginable health state.' A copy of the questionnaire can be found in Appendix 3.

5.3.2.3. Short Form-36 Health Survey (SF-36)

The SF-36 is widely used 36-item questionnaire that measures general health-related quality of life in the following eight domains: physical function, role limitations due to physical health, bodily pain, general health perception, vitality, social functioning, role limitation due to emotional problems, and mental health. Scores for the 8 domains range from 0-100 where higher scores are better. Two additional overall summary scores – physical and mental component scores - will also be obtained. Summary scores are standardized where the general population mean is 50. A copy of the questionnaire can be found in Appendix 4.

5.3.3. Safety Assessments

5.3.3.1. Targeted Medical History

Patient medical history of injection site reactions, infusion reactions, serum sickness, and thromboembolic events should be recorded.

5.3.4. Drug Concentration and Antidrug Antibody Assessments

Any concentration lacking date and time of collection, date of previous dose administration, or patient identification, will not be included in the therapeutic drug analyses. Any concentration reported below the limit of quantitation will be assumed to be zero.

5.3.4.1. Serum for Analysis of Etanercept, Adalimumab, and Infliximab

Blood samples for drug concentration analysis will be collected into appropriately labeled tubes at the assessment visit in order to analyze concentrations of etanercept, adalimumab, or infliximab, depending on the current anti-TNF biologic drug treatment.

Detailed collection, processing, storage, and shipment instructions are provided in the laboratory manual. Samples will be analyzed using validated enzyme-linked immunosorbent assay (ELISA) analytical methods.[8,13]

090177e184841661\Approved\Approved On: 09-Sep-2013 19:01

090177e184841661\Approved\Approved On: 09-Sep-2013 19:01

5.3.4.2. Serum for Analysis of Immunogenicity: Etanercept, Adalimumab, and Infliximab Antidrug Antibody

Blood samples for antidrug antibody analysis will be collected into appropriately labeled tubes at the assessment visit, in order to test for antidrug antibodies for etanercept, adalimumab, or infliximab, depending on the current anti-TNF biologic drug treatment.

Detailed collection, processing, storage, and shipment instructions are provided in the laboratory manual. Samples will be analyzed using validated radioimmunoassay (RIA) analytical methods.[14]

5.4. Study Size

A total of 600 patients will be included in this study (200 treated being treated with etanercept, 200 with adalimumab, and 200 with infliximab). This sample is enough to provide >95% power to detect a difference of 12% in the proportion of patients positive for antidrug antibodies between the group of patients treated with a soluble receptor TNF inhibitor (etanercept) and the group of patients treated with monoclonal antibody TNF inhibitors (adalimumab and infliximab) (5% vs 17%, respectively), using a Chi-squared test with continuity correction, an alpha of 0.05, and attrition rate of 15%.

Sample size was determined based on the primary endpoint. However, the sample size is also large enough for the analysis of secondary endpoints 1 and 2, as described in Section 4.2.2 of this document.

5.5. Data Management

5.5.1. Case Report Forms/Electronic Data Record

As used in this study, the term CRF should be understood to refer to an electronic data record, depending on the data collection method used in this study.

A CRF is required and should be completed for each included patient. The completed original CRFs are the sole property of Pfizer and should not be made available in any form to third parties, except for authorized representatives of Pfizer or appropriate regulatory authorities, without written permission from Pfizer.

The investigator has ultimate responsibility for the collection and reporting of all clinical, safety, and laboratory data entered on the CRFs and any other data collection forms (source documents) and ensuring that they are accurate, authentic/original, attributable, complete, consistent, legible, timely (contemporaneous), enduring, and available when required. The CRFs must be signed by the investigator or by an authorized staff member to attest that the data contained on the CRFs is true. Any corrections to entries made in the CRFs, source documents must be dated, initialed and explained (if necessary), and should not obscure the original entry.

In most cases, the source documents are the hospital's or the physician's patient chart. In these cases data collected on the CRFs must match the data in those charts.

In some cases, the CRF, or part of the CRF, may also serve as source documents. In these cases, a document should be available at the investigator's site as well as at Pfizer and clearly identify those data that will be recorded in the CRF, and for which the CRF will stand as the source document.

5.5.2. Record Retention

To enable evaluations and/or audits from regulatory authorities or Pfizer, the investigator agrees to keep records, including the identity of all participating patients (sufficient information to link records, eg, CRFs and hospital records), all original signed informed consent documents, copies of all CRFs, safety reporting forms, source documents, and detailed records of treatment disposition, and adequate documentation of relevant correspondence (eg, letters, meeting minutes, telephone calls reports). The records should be retained by the investigator according to International Conference on Harmonisation (ICH), local regulations, or as specified in the Clinical Study Agreement (CSA), whichever is longer.

If the investigator becomes unable for any reason to continue to retain study records for the required period (eg, retirement, relocation), Pfizer should be prospectively notified. The study records must be transferred to a designee acceptable to Pfizer, such as another investigator, another institution, or to an independent third party arranged by Pfizer. Investigator records must be kept for a minimum of 15 years after completion or discontinuation of the study or for longer if required by applicable local regulations.

The investigator must obtain Pfizer's written permission before disposing of any records, even if retention requirements have been met.

5.6. Data Analysis

Detailed methodology for summary and statistical analyses of data collected in this study will be documented in a Statistical Analysis Plan (SAP), which will be dated, filed and maintained by the sponsor. The SAP may modify the plans outlined in the protocol; any major modifications of primary endpoint definitions or their analyses would be reflected in a protocol amendment. The analysis of the primary endpoint (the proportion of patients positive for antidrug antibodies) will use a logistic regression model with terms for treatment currently being received by patient (etanercept, adalimumab/infliximab) and other co-variates to adjust for imbalances between groups in potential confounding factors, including such as:

- Duration of treatment.
- Concomitant use of methotrexate.
- Frequency and dose of etanercept/adalimumab/infliximab.

Secondary endpoints will be comprised by two types of analyses:

- The comparison between the proportions of patients with certain characteristics in the subset of patients with antidrug antibody positive versus those with antidrug antibody

090177e184841661\Approved\Approved On: 09-Sep-2013 19:01

negative will be performed using a Chi-squared test (or a Fisher's exact test). This analysis will be performed for all patients combined.

- The comparison between continuous variables (such as serum trough drug concentration, efficacy scores, etc) in the subset of patients with antibody positive versus those with antibody negative will be performed using t-tests, if the distribution is found to follow a normal distribution. Non-parametric tests will be used if the distribution of serum trough drug concentration departs from normality. The analysis of serum trough drug concentration will only be performed if there is an adequate amount of data.

Secondary endpoints and exploratory endpoints will be analyzed using the same approach as described for the first secondary endpoints above. Continuous variables will be compared between subset of patients with positive versus those with negative antibody using t-tests and non-parametric tests in case of non-normal distribution; whereas categorical variables will be analyzed using Chi-squared and Fisher's exact tests. Correlation between two continuous variables will be represented by Pearson's and Spearman's correlation coefficient, along with scatter plots.

Secondary endpoints related to serum trough drug concentration (secondary endpoints numbered 2, 3, and 7 and exploratory endpoints numbered 3, 6, and 8) will be analyzed separately by the type of treatment patients are currently taking (etanercept, adalimumab or infliximab). Secondary endpoints numbered 4, 5 and 6 and exploratory endpoints numbered 1, 2, 4, 5, and 7 will be analyzed by the type of treatment and also for the overall combined sample.

5.7. Quality Control

During study conduct, Pfizer or its agent will conduct periodic monitoring visits to ensure that the protocol is being followed. The monitors may review source documents to confirm that the data recorded on CRFs is accurate. The investigator and institution will allow Pfizer monitors or its agents and appropriate regulatory authorities direct access to source documents to perform this verification.

The study site may be subject to review by the Institutional Review Board (IRB)/Independent Ethics Committee (IEC), and/or to quality assurance audits performed by Pfizer, or companies working with or on behalf of Pfizer, and/or to inspection by appropriate regulatory authorities.

It is important that the investigator(s) and their relevant personnel are available during the monitoring visits and possible audits or inspections and that sufficient time is devoted to the process.

090177e184841661\Approved\Approved On: 09-Sep-2013 19:01

090177e184841661\Approved\Approved On: 09-Sep-2013 19:01

6. PROTECTION OF HUMAN SUBJECTS

6.1. Patient Information and Consent

All parties will ensure protection of patient personal data and will not include patient names on any sponsor forms, reports, publications, or in any other disclosures, except where required by laws. In case of data transfer, Pfizer will maintain high standards of confidentiality and protection of patient personal data.

The informed consent form must be in compliance with local regulatory requirements and legal requirements.

The informed consent form used in this study, and any changes made during the course of the study, must be prospectively approved by both the IRB/IEC and Pfizer before use.

The investigator must ensure that each study patient, or his/her legally acceptable representative, is fully informed about the nature and objectives of the study and possible risks associated with participation. The investigator, or a person designated by the investigator, will obtain written informed consent from each patient or the patient's legally acceptable representative before any study-specific activity is performed. The investigator will retain the original of each patient's signed consent form.

6.2. Patient Withdrawal

Patients may withdraw from the study at any time at their own request, or they may be withdrawn at any time at the discretion of the investigator or sponsor for safety, behavioral, or administrative reasons. In any circumstance, every effort should be made to document patient outcome, if possible. The investigator should inquire about the reason for withdrawal and follow-up with the patient regarding any unresolved adverse events.

If the patient withdraws from the study, and also withdraws consent for disclosure of future information, no further evaluations should be performed, and no additional data should be collected. The sponsor may retain and continue to use any data collected before such withdrawal of consent.

6.3. Institutional Review Board (IRB)/Independent Ethics Committee (IEC)

It is the responsibility of the investigator to have prospective approval of the study protocol, protocol amendments, and informed consent forms, and other relevant documents, (eg, recruitment advertisements), if applicable, from the IRB/IEC. All correspondence with the IRB/IEC should be retained in the Investigator File. Copies of IRB/IEC approvals should be forwarded to Pfizer.

6.4. Ethical Conduct of the Study

The study will be conducted in accordance with legal and regulatory requirements, as well as with scientific purpose, value and rigor and follow generally accepted research practices described in *Good Pharmacoepidemiology Practices* (GPP) issued by the International Society for Pharmacoepidemiology (ISPE), Good Epidemiological Practice (GEP) guidelines

issued by the International Epidemiological Association (IEA), Good Outcomes Research Practices issued by the International Society for Pharmacoeconomics and Outcomes Research (ISPOR), International Ethical Guidelines for Epidemiological Research issued by the Council for International Organizations of Medical Sciences (CIOMS), European Medicines Agency (EMA) European Network of Centres for Pharmacoepidemiology and Pharmacovigilance (ENCePP) Guide on Methodological Standards in Pharmacoepidemiology, and FDA Guidance for Industry: Good Pharmacovigilance and Pharmacoepidemiologic Assessment, FDA Draft Guidance for Industry and FDA Staff: Best Practices for Conducting and Reporting of Pharmacoepidemiologic Safety Studies Using Electronic Healthcare Data Sets, Guidance for Industry: Patient-Reported Outcome Measures: Use in Medical Product Development to Support Labeling Claims and/or equivalent.

7. ADVERSE EVENT REPORTING

7.1. Adverse Events

All observed or volunteered adverse events regardless of treatment group (if applicable) or suspected causal relationship to etanercept, adalimumab, or infliximab will be recorded on the adverse event page(s) of the CRF as follows.

For all adverse events, the investigator must pursue and obtain information adequate both to determine the outcome of the adverse event and to assess whether it meets the criteria for classification as a serious adverse event (see section "Serious Adverse Events") requiring immediate notification to Pfizer or a Pfizer-designated representative. For all adverse events, sufficient information should be obtained by the investigator to determine the causality of the adverse event. The investigator is required to assess causality. For adverse events with a causal relationship to etanercept, adalimumab, or infliximab, follow-up by the investigator is required until the event or its sequelae resolve or stabilize at a level acceptable to the investigator, and Pfizer concurs with that assessment.

7.2. Reporting Period

For non-serious and serious adverse events, the reporting period to Pfizer or its designated representative begins from the time of the patient's first dosing in the observational period as per study design through and including 28 calendar days after the last administration of the study drug within the observational period. If the investigator becomes aware of a SAE that is considered related to study drug occurring at any other time after completion of the study, the SAE is also reportable.

7.3. Definition of Adverse Event

An AE is any untoward medical occurrence in a patient administered a medicinal or nutritional product (including infant and toddler formulas [hereinafter "pediatric formulas"]) or medical device. The event need not necessarily have a causal relationship with the product treatment or usage. Examples of adverse events include but are not limited to:

- Abnormal test findings;

090177e184841661\Approved\Approved On: 09-Sep-2013 19:01

- Clinically significant symptoms and signs;
- Changes in physical examination findings;
- Hypersensitivity;
- Progression/worsening of underlying disease;
- Lack of efficacy;
- Drug abuse;
- Drug dependency.

Additionally, for medicinal products, they may include the signs or symptoms resulting from:

- Drug overdose;
- Drug withdrawal;
- Drug misuse;
- Off-label use;
- Drug interactions;
- Extravasation;
- Exposure during pregnancy;
- Exposure during breast feeding;
- Medication error;
- Occupational exposure.

7.4. Abnormal Test Findings

The criteria for determining whether an abnormal objective test finding should be reported as an adverse event are as follows:

- Test result is associated with accompanying symptoms, and/or
- Test result requires additional diagnostic testing or medical/surgical intervention, and/or
- Test result leads to a change in study dosing or discontinuation from the study, significant additional concomitant drug treatment, or other therapy, and/or

090177e184841661\Approved\Approved On: 09-Sep-2013 19:01

- Test result is considered to be an adverse event by the investigator or sponsor.

Merely repeating an abnormal test, in the absence of any of the above conditions, does not constitute an adverse event. Any abnormal test result that is determined to be an error does not require reporting as an adverse event.

7.5. Serious Adverse Events

A serious adverse event is any untoward medical occurrence in a patient administered a medicinal or nutritional product (including pediatric formulas) at any dose that:

- Results in death;
- Is life-threatening;
- Requires inpatient hospitalization or prolongation of hospitalization;
- Results in persistent or significant disability/incapacity (substantial disruption of the ability to conduct normal life functions);
- Results in congenital anomaly/birth defect.

Lack of efficacy should be reported as an adverse event when it is associated with a serious adverse event.

Medical and scientific judgment is exercised in determining whether an event is an important medical event. An important medical event may not be immediately life-threatening and/or result in death or hospitalization. However, if it is determined that the event may jeopardize the patient or may require intervention to prevent one of the other outcomes listed in the definition above, the important medical event should be reported as serious.

Examples of such events are intensive treatment in an emergency room or at home for allergic bronchospasm; blood dyscrasias or convulsions that do not result in hospitalization; or development of drug dependency or drug abuse.

Additionally, any suspected transmission via a Pfizer product of an infectious agent, pathogenic or non-pathogenic, is considered serious. The event may be suspected from clinical symptoms or laboratory findings indicating an infection in a patient exposed to a Pfizer product. The terms “suspected transmission” and “transmission” are considered synonymous. These cases are considered unexpected and handled as serious expedited cases by PV personnel. Such cases are also considered for reporting as product defects, if appropriate.

7.6. Hospitalization

Adverse events reported from studies associated with hospitalization or prolongation of hospitalization are considered serious. Any initial admission (even if less than 24 hours) to a healthcare facility meets these criteria. Admission also includes transfer within the hospital

090177e184841661\Approved\Approved On: 09-Sep-2013 19:01

to an acute/intensive care unit (eg, from the psychiatric wing to a medical floor, medical floor to a coronary care unit, neurological floor to a tuberculosis unit).

Hospitalization in the absence of a medical AE is not in itself an AE and is not reportable. For example, the following reports of hospitalization without a medical AE are not to be reported.

- Social admission (eg, patient has no place to sleep);
- Administrative admission (eg, for yearly exam);
- Optional admission not associated with a precipitating medical AE (eg, for elective cosmetic surgery);
- Hospitalization for observation without a medical AE;
- Admission for treatment of a pre-existing condition not associated with the development of a new AE or with a worsening of the pre-existing condition (eg, for work-up of persistent pre-treatment lab abnormality);
- Protocol-specified admission during clinical study (eg, for a procedure required by the study protocol).

7.7. Causality Assessment

The investigator's assessment of causality must be provided for all adverse events (serious and non-serious). The investigator must record the causal relationship in the CRF, as appropriate, and report such an assessment in accordance with the serious adverse reporting requirements if applicable.

An investigator's causality assessment is the determination of whether there exists a reasonable possibility that etanercept, adalimumab, or infliximab caused or contributed to an adverse event. If the investigator's final determination of causality is unknown and the investigator does not know whether etanercept, adalimumab, or infliximab caused the event, then the event will be handled as related to etanercept, adalimumab, or infliximab for reporting purposes. If the investigator's causality assessment is unknown but not related to etanercept, adalimumab, or infliximab this should be clearly documented in the CRF.

7.8. Exposure During Pregnancy

An exposure during pregnancy (also referred to as exposure in-utero [EIU]) occurs if:

1. A female becomes, or is found to be, pregnant either while receiving or having been exposed to (eg, environmental) etanercept, adalimumab, or infliximab, or the female becomes, or is found to be, pregnant after discontinuing and/or being exposed to etanercept, adalimumab, or infliximab (maternal exposure).

090177e184841661\Approved\Approved On: 09-Sep-2013 19:01

2. A male has been exposed, either due to treatment or environmental exposure to etanercept, adalimumab, or infliximab prior to or around the time of conception and/or is exposed during the partner pregnancy (paternal exposure).

As a general rule, prospective and retrospective exposure during pregnancy reports from any source are reportable irrespective of the presence of an associated AE/SAE.

An example of environmental exposure would be a case involving direct contact with a Pfizer product in a pregnant woman (eg, a nurse reports that she is pregnant and has been exposed to chemotherapeutic products).

If a study patient or study patient's partner becomes, or is found to be, pregnant during the study patient's treatment with etanercept, adalimumab, or infliximab, the investigator must submit this information to Pfizer within 24 hours of awareness of the pregnancy, irrespective of whether an adverse event has occurred.

Follow-up is conducted to obtain pregnancy outcome information on all Exposure in Utero reports with an unknown outcome. The investigator will follow the pregnancy until completion or until pregnancy termination (eg, induced abortion) and then notify Pfizer of the outcome. The investigator will provide this information as a follow up to the initial Exposure in Utero report.

For clinical studies conducted in pregnant women, data on the pregnancy outcome and non-serious AEs are expected to be collected and analyzed in the clinical database. In such instances only EIUs associated with a SAE are to be reported.

7.9. Medication Error

A medication error is any unintentional error in the prescribing, dispensing or administration of a medicinal product that may cause or lead to inappropriate medication use or patient harm while in the control of the health care professional, patient, or consumer. Such events may be related to professional practice, health care products, procedures, and systems including: prescribing; order communication; product labeling, packaging, and nomenclature; compounding; dispensing; distribution; administration; education; monitoring; and use.

Medication errors include:

- Near misses, involving or not involving a patient directly (eg, inadvertent/erroneous administration, which is the accidental use of a product outside of labeling or prescription on the part of the healthcare provider or the patient/consumer);
- Confusion with regard to invented name (eg, trade name, brand name).

The investigator must submit the following medication errors to Pfizer within 24 hours of awareness, irrespective of the presence of an associated AE/SAE:

090177e184841661\Approved\Approved On: 09-Sep-2013 19:01

- Medication errors involving patient exposure to the product, whether or not the medication error is accompanied by an AE.
- Medication errors that do not involve a patient directly (eg, potential medication errors or near misses). When a medication error does not involve patient exposure to the product the following minimum criteria constitute a medication error report:
  - An identifiable reporter;
  - A suspect product;
  - The event medication error.

7.10. Reporting Requirements

Each adverse event is to be assessed to determine if it meets the criteria for serious adverse events.

If a serious adverse event occurs, expedited reporting will follow local and international regulations, as appropriate.

7.11. Serious or Non-Serious Adverse Event Reporting Requirements

If a serious or non-serious adverse event occurs, Pfizer is to be notified within 24 hours of awareness of the event by the investigator. In particular, if the serious adverse event is fatal or life-threatening, notification to Pfizer must be made immediately, irrespective of the extent of available adverse event information. This timeframe also applies to additional new information (follow-up) on previously forwarded serious or non-serious adverse event reports as well as to the initial and follow-up reporting of exposure during pregnancy, exposure during breast feeding and medication error cases.

In the rare event that the investigator does not become aware of the occurrence of a serious or non-serious adverse event immediately (eg, if an outpatient study patient initially seeks treatment elsewhere), the investigator is to report the event within 24 hours after learning of it and document the time of his/her first awareness of the adverse event.

For all serious or non-serious adverse events, the investigator is obligated to pursue and provide information to Pfizer in accordance with the timeframes for reporting specified above. In addition, an investigator may be requested by Pfizer to obtain specific additional follow-up information in an expedited fashion. This information collected for serious or non-serious adverse events is more detailed than that captured on the adverse event case report form. In general, this will include a description of the adverse event in sufficient detail to allow for a complete medical assessment of the case and independent determination of possible causality. Information on other possible causes of the event, such as concomitant medications and illnesses must be provided. In the case of a patient death, a summary of available autopsy findings must be submitted as soon as possible to Pfizer or its designated representative.

090177e184841661\Approved\Approved On: 09-Sep-2013 19:01

090177e184841661\Approved\Approved On: 09-Sep-2013 19:01

7.12. Single Reference Safety Document

Complete safety information for etanercept may be found in the Single Reference Safety Document, which for this study is the Enbrel Core Data Sheet. The Single Reference Safety Document for adalimumab and infliximab would be the most recent version of their respective US Product Labels.

8. PLANS FOR DISSEMINATING AND COMMUNICATING STUDY RESULTS

8.1. Communication of Results by Pfizer

Pfizer fulfills its commitment to publicly disclose clinical trial results through posting the results of this study on www.clinicaltrials.gov (ClinicalTrials.gov). Pfizer posts the results of all studies that it has registered on ClinicalTrials.gov regardless of the reason for registration.

The results are posted in a tabular format called Basic Results.

For studies involving a Pfizer product, the timing of the posting depends on whether the Pfizer product is approved for marketing in any country at the time the study is completed:

- For studies involving products applicable under the US Food and Drug Administration Amendments Act of 2007 (FDAAA), ie, FDA-approved products, Pfizer posts results within one year of the primary outcome completion date (PCD). For studies involving products approved in any country, but not FDA approved, Pfizer posts results one year from last subject, last visit (LSLV).

- For studies involving products that are not yet approved in any country, Pfizer posts the results of already-completed studies within 30 days of US regulatory approval, or one year after the first ex-US regulatory approval of the product (if only submitted for approval ex-US).

- For studies involving products whose drug development is discontinued before approval, Pfizer posts the results within one year of discontinuation of the program (if there are no plans for outlicensing or within two years if outlicensing plans have not completed).

Primary Completion Date is defined as the date that the final patient was examined for the purposes of final collection of data for the primary outcome, whether the clinical trial concluded according to the pre-specified protocol or was terminated.

8.2. Publications by Investigators

Pfizer has no objection to publication by Investigator of any information collected or generated by Investigator, whether or not the results are favorable to the Investigational Drug. However, to ensure against inadvertent disclosure of Confidential Information or unprotected Inventions, Investigator will provide Pfizer an opportunity to review any proposed publication or other type of disclosure before it is submitted or otherwise disclosed.

Investigator will provide manuscripts, abstracts, or the full text of any other intended disclosure (poster presentation, invited speaker or guest lecturer presentation, etc.) to Pfizer at least 30 days before they are submitted for publication or otherwise disclosed. If any patent action is required to protect intellectual property rights, Investigator agrees to delay the disclosure for a period not to exceed an additional 60 days.

Investigator will, on request, remove any previously undisclosed Confidential Information (other than the Study results themselves) before disclosure.

If the Study is part of a multi-centre study, Investigator agrees that the first publication is to be a joint publication covering all centers. However, if a joint manuscript has not been submitted for publication within 12 months of completion or termination of the Study at all participating sites, Investigator is free to publish separately, subject to the other requirements of this Section.

For all publications relating to the Study, Institution will comply with recognized ethical standards concerning publications and authorship, including Section II - “Ethical Considerations in the Conduct and Reporting of Research” of the Uniform Requirements for Manuscripts Submitted to Biomedical Journals, http://www.icmje.org/index.html#authorship, established by the International Committee of Medical Journal Editors.

Publication of study results is also provided for in the Clinical Study Agreement between Pfizer and the institution. In this section entitled Publications by Investigators, the defined terms shall have the meanings given to them in the Clinical Study Agreement.

090177e184841661\Approved\Approved On: 09-Sep-2013 19:01

9. REFERENCES

1. Tracey D, Klareskog L, Sasso EH, et al. Tumor necrosis factor antagonist mechanisms of action: A comprehensive review. *Pharmacol Ther.* 2008;117:244-279.

2. Krieckaert CLM, Bartelds G, Lems W, et al. The effect of immunomodulators on the immunogenicity of TNF-blocking therapeutic monoclonal antibodies: a review. *Arthritis Res Ther*. 2010;12:217-223.

3. Pascual-Salcedo D, Plasencia C, Ramiro S, et al. Influence of immunogenicity on the efficacy of long term treatment with infliximab in rheumatoid arthritis. *Rheumatology.* 2011;50:1445-1452.

4. Radstake TRDJ, Svenson M, Eijsbouts AM, et al. Formation of antibodies against infliximab and adalimumab strongly correlates with functional drug levels and clinical responses in rheumatoid arthritis. *Ann Rheum Dis.* 2009;68:1739-1745.

5. Korswagen LA, Bartelds G, Krieckaert CLM, et al. Venous and arterial thromboembolic events in adalimumab-treated patients with antiadalimumab antibodies. *Arthritis and Rheumatism*. 2011;63:877-883.

6. Bendtzen K, Geborek P, Svenson M, et al. Individualized monitoring of drug bioavailability and immunogenicity in rheumatoid arthritis patients treated with the tumor necrosis factor alpha inhibitor infliximab. *Arthritis Rheum.* 2006;54(12):3782-3789.

7. Finckh A, Dudler J, Wermelinger F, et al. Influence of anti-infliximab antibodies and residual infliximab concentrations on the occurrence of acquired drug resistance to infliximab in rheumatoid arthritis patients. *Joint Bone Spine.* 2010;77(4):313-318.

8. Bartelds G, Krieckaert C, Nurmohamed MT, et al. Development of antidrug antibodies against adalimumab and association with disease activity and treatment failure during long-term follow-up. *JAMA.* 2011;305(14):1460-1468.

9. Dore RK, Mathews S, Schechtman W, et al. The immunogenicity, safety, and efficacy of etanercept liquid administered once weekly in patients with rheumatoid arthritis. *Clin Exp Rheumatol*. 2007;25:40-46.

10. Anderson PJ. Tumor necrosis factor inhibitors: Clinical implications of their different immunogenicity profiles. *Semin Arthritis Rheum*. 2005;34(Suppl 1):19-22.

11. Prevoo ML, van ‘t Hof, Kuper HH, et al. Modified disease activity scores that include twenty-eight-joint counts. *Arthritis & Rheumatism.* 1995;38:44-48.

12. Aletaha D, Smolen J. The Simplified Disease Activity Index (SDAI) and the Clinical Disease Activity Index (CDAI): A review of their usefulness and validity in rheumatoid arthritis. *Clin Exp Rheumatol.* 2005;23(39):S100-S109.

090177e184841661\Approved\Approved On: 09-Sep-2013 19:01

13. Wolbink GJ, Voskuyl AE, Lems WF, et al. Relationship between serum trough infliximab levels, pretreatment C reactive protein levels, and clinical response to infliximab treatment in patients with rheumatoid arthritis. *Ann Rheum Dis.* 2005;64:704-707.

14. Aarden L, Ruuls SR, Wolbink G. Immunogenicity of anti-tumor necrosis factor antibodies – toward improved methods of anti-antibody measurement. *Current Opinion in Immunology.* 2008; 20:431-435.

10. LIST OF FIGURES

Figure 1. Study Schematic

090177e184841661\Approved\Approved On: 09-Sep-2013 19:01

11. APPENDICES

Appendix 1. 1987 Revised Criteria for the Classification of Rheumatoid Arthritis

1987 Revised Criteria for the Classification of Rheumatoid Arthritis	
1. Morning stiffness	Morning stiffness in and around the joints, lasting at least 1 hour before maximal improvement
2. Arthritis of 3 or more joint areas	At least 3 joint areas simultaneously have had soft tissue swelling or fluid (not bony overgrowth alone) observed by a physician. The 14 possible areas are right or left PIP, MCP, wrist, elbow, knee, ankle, and MTP joints
3. Arthritis of hand joints	At least 1 area swollen (as defined above) in a wrist, MCP, or PIP joint
4. Symmetric arthritis	Simultaneous involvement of the same joint areas (as defined in 2) on both sides fo the body (bilateral involvement of PIPs, MCPs, or MTPs is acceptable without absolute symmetry)
5. Rheumatoid nodules	Subcutaneous nodules, over bony prominences, or extensor surfaces, or in juxtaarticular regions, observed by a physician
6. Serum rheumatoid factor	Demonstration of abnormal amounts of serum rheumatoid factor by any method for which the result has been positive in <5% of normal control subjects
7. Radiographic changes	Radiographic changes typical of rheumatoid arthritis on posteroanterior hand and wrist radiographs, which must include erosions or unequivocal bony decalcification localized in or most marked adjacent to the involved joints (osteoarthritis changes alone do not qualify)
For classification purposes, a patient shall be said to have rheumatoid arthritis if he/she has satisfied at least 4 of these 7 criteria. Criteria 1 through 4 must have been present for at least 6 weeks. Patients with 2 clinical diagnoses are not excluded. Designation as classic, definite, or probable rheumatoid arthritis is not to be made.<br>Arnett FC, Edworthy SM, Bloch DA, McShane DJ, Fries JF, Cooper NS, et al. The American Rheumatism Association 1987 revised criteria for the classification of rheumatoid arthritis. Arthritis Rheum 1988;31:315--24.	

090177e184841661\Approved\Approved On: 09-Sep-2013 19:01

Appendix 2. Sample Health Assessment Questionnaire – Disability Index

HEALTH ASSESSMENT QUESTIONAIRE

In this section we are interested in learning how your illness affects your ability to function in daily life.

Please check the response which best describes your usual abilities OVER THE PAST WEEK:

	Without ANY Difficulty	With SOME Difficulty	With MUCH Difficulty	UNABLE To Do
**DRESSING & GROOMING**				
Are you able to:				
- Dress yourself, including tying shoelaces and doing buttons?	☐	☐	☐	☐
- Shampoo your hair?	☐	☐	☐	☐
**ARISING**				
Are you able to:				
- Stand up from a straight chair?	☐	☐	☐	☐
- Get in and out of bed?	☐	☐	☐	☐
**EATING**				
Are you able to:				
- Cut your meat?	☐	☐	☐	☐
- Lift a full cup or glass to your mouth?	☐	☐	☐	☐
- Open a new milk carton?	☐	☐	☐	☐
**WALKING**				
Are you able to:				
- Walk outdoors on flat ground?	☐	☐	☐	☐
- Climb up five steps?	☐	☐	☐	☐

Please check any AIDS OR DEVICES that you usually use for any of these activities:

- ☐ Cane
- ☐ Walker
- ☐ Crutches
- ☐ Wheelchair
- ☐ Devices used for dressing (button hook, zipper pull, long-handled shoe horn, etc.)
- ☐ Built up or special utensils
- ☐ Special or built up chair
- ☐ Other (Specify: ______________________)

Please check any categories for which you usually need HELP FROM ANOTHER PERSON:

- ☐ Dressing and Grooming
- ☐ Arising
- ☐ Eating
- ☐ Walking

Sample

090177e184841661\Approved\Approved On: 09-Sep-2013 19:01

Please check the response which best describes your usual abilities OVER THE PAST WEEK:

	Without ANY Difficulty	With SOME Difficulty	With MUCH Difficulty	UNABLE To Do
**HYGIENE**				
Are you able to:				
- Wash and dry your body?	☐	☐	☐	☐
- Take a tub bath?	☐	☐	☐	☐
- Get on and off the toilet?	☐	☐	☐	☐
**REACH**				
Are you able to:				
- Reach and get down a 5 pound object (such as a bag of sugar) from just above your head?	☐	☐	☐	☐
- Bend down to pick up clothing from the floor?	☐	☐	☐	☐
**GRIP**				
Are you able to:				
- Open car doors?	☐	☐	☐	☐
- Open jars which have been previously opened?	☐	☐	☐	☐
- Turn faucets on and off?	☐	☐	☐	☐
**ACTIVITIES**				
Are you able to:				
- Run errands and shop?	☐	☐	☐	☐
- Get in and out of a car?	☐	☐	☐	☐
- Do chores such as vacuuming or yardwork?	☐	☐	☐	☐

Please check any AIDS OR DEVICES that you usually use for any of these activities:

☐ Raised toilet seat
☐ Bathtub seat
☐ Jar opener (for jars previously opened)
☐ Bathtub bar
☐ Long-handled appliances for reach
☐ Long-handled appliances in bathroom
☐ Other (Specify: ______________________)

Please check any categories for which you usually need HELP FROM ANOTHER PERSON:

☐ Hygiene
☐ Reach
☐ Gripping and opening things
☐ Errands and chores

Reproduced with permission of Stanford University Copyright © MAY 99

Sample

090177e184841661\Approved\Approved On: 09-Sep-2013 19:01

Appendix 3. Sample EQ-5D Questionnaire

EQ-5D

Health Questionnaire

(English version for the US)

Sample

© 1998 EuroQol Group. EQ-5D™ is a trade mark of the EuroQol Group

090177e184841661\Approved\Approved On: 09-Sep-2013 19:01

By placing a checkmark in one box in each group below, please indicate which statements best describe your own health state today.

Mobility

I have no problems in walking about ❑

I have some problems in walking about ❑

I am confined to bed ❑

Self-Care

I have no problems with self-care ❑

I have some problems washing or dressing myself ❑

I am unable to wash or dress myself ❑

Usual Activities (*e.g. work, study, housework, family or leisure activities)*

I have no problems with performing my usual activities ❑

I have some problems with performing my usual activities ❑

I am unable to perform my usual activities ❑

Pain/Discomfort

I have no pain or discomfort ❑

I have moderate pain or discomfort ❑

I have extreme pain or discomfort ❑

Anxiety/Depression

I am not anxious or depressed ❑

I am moderately anxious or depressed ❑

I am extremely anxious or depressed ❑

2

© 1998 EuroQol Group. EQ-5D™ is a trade mark of the EuroQol Group

Sample

090177e184841661\Approved\Approved On: 09-Sep-2013 19:01

To help people say how good or bad a health state is, we have drawn a scale (rather like a thermometer) on which the best state you can imagine is marked 100 and the worst state you can imagine is marked 0.

We would like you to indicate on this scale how good or bad your own health is today, in your opinion. Please do this by drawing a line from the box below to whichever point on the scale indicates how good or bad your health state is today.

Your own health state today

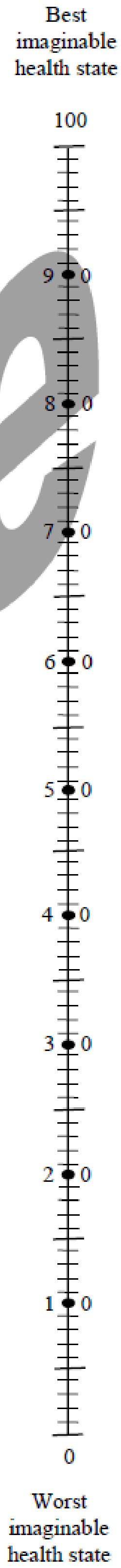

Sample

3

© 1998 EuroQol Group. EQ-5D™ is a trade mark of the EuroQol Group

090177e184841661\Approved\Approved On: 09-Sep-2013 19:01

Appendix 4. Sample 36-Item Short-Form Health Survey

Your Health and Well-Being

This survey asks for your views about your health. This information will help keep track of how you feel and how well you are able to do your usual activities. ***Thank you for completing this survey!***

For each of the following questions, please mark an ☒ in the one box that best describes your answer.

1. In general, would you say your health is:

Excellent	Very good	Good	Fair	Poor
☐ 1	☐ 2	☐ 3	☐ 4	☐ 5

2. <u>Compared to one year ago</u>, how would you rate your health in general <u>now</u>?

Much better now than one year ago	Somewhat better now than one year ago	About the same as one year ago	Somewhat worse now than one year ago	Much worse now than one year ago
☐ 1	☐ 2	☐ 3	☐ 4	☐ 5

SF-36v2® Health Survey © 1992, 1996, 2000 Medical Outcomes Trust and QualityMetric Incorporated. All rights reserved.
SF-36® is a registered trademark of Medical Outcomes Trust.
(SF-36v2® Health Survey Standard, United States (English))

Sample

090177e184841661\Approved\Approved On: 09-Sep-2013 19:01

3. The following questions are about activities you might do during a typical day. Does your health now limit you in these activities? If so, how much?

	Yes, limited a lot	Yes, limited a little	No, not limited at all
a Vigorous activities, such as running, lifting heavy objects, participating in strenuous sports	☐ 1	☐ 2	☐ 3
b Moderate activities, such as moving a table, pushing a vacuum cleaner, bowling, or playing golf	☐ 1	☐ 2	☐ 3
c Lifting or carrying groceries	☐ 1	☐ 2	☐ 3
d Climbing several flights of stairs	☐ 1	☐ 2	☐ 3
e Climbing one flight of stairs	☐ 1	☐ 2	☐ 3
f Bending, kneeling, or stooping	☐ 1	☐ 2	☐ 3
g Walking more than a mile	☐ 1	☐ 2	☐ 3
h Walking several hundred yards	☐ 1	☐ 2	☐ 3
i Walking one hundred yards	☐ 1	☐ 2	☐ 3
j Bathing or dressing yourself	☐ 1	☐ 2	☐ 3

Sample

SF-36v2® Health Survey © 1992, 1996, 2000 Medical Outcomes Trust and QualityMetric Incorporated. All rights reserved.
SF-36® is a registered trademark of Medical Outcomes Trust.
(SF-36v2® Health Survey Standard, United States (English))

090177e184841661\Approved\Approved On: 09-Sep-2013 19:01

4. During the past 4 weeks, how much of the time have you had any of the following problems with your work or other regular daily activities as a result of your physical health?

	All of the time	Most of the time	Some of the time	A little of the time	None of the time
a Cut down on the amount of time you spent on work or other activities	☐ 1	☐ 2	☐ 3	☐ 4	☐ 5
b Accomplished less than you would like	☐ 1	☐ 2	☐ 3	☐ 4	☐ 5
c Were limited in the kind of work or other activities	☐ 1	☐ 2	☐ 3	☐ 4	☐ 5
d Had difficulty performing the work or other activities (for example, it took extra effort)	☐ 1	☐ 2	☐ 3	☐ 4	☐ 5

5. During the past 4 weeks, how much of the time have you had any of the following problems with your work or other regular daily activities as a result of any emotional problems (such as feeling depressed or anxious)?

	All of the time	Most of the time	Some of the time	A little of the time	None of the time
a Cut down on the amount of time you spent on work or other activities	☐ 1	☐ 2	☐ 3	☐ 4	☐ 5
b Accomplished less than you would like	☐ 1	☐ 2	☐ 3	☐ 4	☐ 5
c Did work or other activities less carefully than usual	☐ 1	☐ 2	☐ 3	☐ 4	☐ 5

sample

SF-36v2® Health Survey © 1992, 1996, 2000 Medical Outcomes Trust and QualityMetric Incorporated. All rights reserved.
SF-36® is a registered trademark of Medical Outcomes Trust.
(SF-36v2® Health Survey Standard, United States (English))

090177e184841661\Approved\Approved On: 09-Sep-2013 19:01

6. During the past 4 weeks, to what extent has your physical health or emotional problems interfered with your normal social activities with family, friends, neighbors, or groups?

Not at all	Slightly	Moderately	Quite a bit	Extremely
☐ 1	☐ 2	☐ 3	☐ 4	☐ 5

7. How much bodily pain have you had during the past 4 weeks?

None	Very mild	Mild	Moderate	Severe	Very severe
☐ 1	☐ 2	☐ 3	☐ 4	☐ 5	☐ 6

8. During the past 4 weeks, how much did pain interfere with your normal work (including both work outside the home and housework)?

Not at all	A little bit	Moderately	Quite a bit	Extremely
☐ 1	☐ 2	☐ 3	☐ 4	☐ 5

Sample

SF-36v2® Health Survey © 1992, 1996, 2000 Medical Outcomes Trust and QualityMetric Incorporated. All rights reserved.
SF-36® is a registered trademark of Medical Outcomes Trust.
(SF-36v2® Health Survey Standard, United States (English))

090177e184841661\Approved\Approved On: 09-Sep-2013 19:01

9. These questions are about how you feel and how things have been with you during the past 4 weeks. For each question, please give the one answer that comes closest to the way you have been feeling. How much of the time during the past 4 weeks…

	All of the time	Most of the time	Some of the time	A little of the time	None of the time
a Did you feel full of life?	☐ 1	☐ 2	☐ 3	☐ 4	☐ 5
b Have you been very nervous?	☐ 1	☐ 2	☐ 3	☐ 4	☐ 5
c Have you felt so down in the dumps that nothing could cheer you up?	☐ 1	☐ 2	☐ 3	☐ 4	☐ 5
d Have you felt calm and peaceful?	☐ 1	☐ 2	☐ 3	☐ 4	☐ 5
e Did you have a lot of energy?	☐ 1	☐ 2	☐ 3	☐ 4	☐ 5
f Have you felt downhearted and depressed?	☐ 1	☐ 2	☐ 3	☐ 4	☐ 5
g Did you feel worn out?	☐ 1	☐ 2	☐ 3	☐ 4	☐ 5
h Have you been happy?	☐ 1	☐ 2	☐ 3	☐ 4	☐ 5
i Did you feel tired?	☐ 1	☐ 2	☐ 3	☐ 4	☐ 5

10. During the past 4 weeks, how much of the time has your physical health or emotional problems interfered with your social activities (like visiting with friends, relatives, etc.)?

All of the time	Most of the time	Some of the time	A little of the time	None of the time
☐ 1	☐ 2	☐ 3	☐ 4	☐ 5

SF-36v2® Health Survey © 1992, 1996, 2000 Medical Outcomes Trust and QualityMetric Incorporated. All rights reserved.
SF-36® is a registered trademark of Medical Outcomes Trust.
(SF-36v2® Health Survey Standard, United States (English))

Sample

090177e184841661\Approved\Approved On: 09-Sep-2013 19:01

11. How TRUE or FALSE is each of the following statements for you?

	Definitely true	Mostly true	Don't know	Mostly false	Definitely false
a I seem to get sick a little easier than other people	☐ 1	☐ 2	☐ 3	☐ 4	☐ 5
b I am as healthy as anybody I know	☐ 1	☐ 2	☐ 3	☐ 4	☐ 5
c I expect my health to get worse	☐ 1	☐ 2	☐ 3	☐ 4	☐ 5
d My health is excellent	☐ 1	☐ 2	☐ 3	☐ 4	☐ 5

Thank you for completing these questions!

Sample

SF-36v2® Health Survey © 1992, 1996, 2000 Medical Outcomes Trust and QualityMetric Incorporated. All rights reserved.
SF-36® is a registered trademark of Medical Outcomes Trust.
(SF-36v2® Health Survey Standard, United States (English))

090177e184841661\Approved\Approved On: 09-Sep-2013 19:01
